# Thermally stable metal–organic framework based iron 2,6-naphthalenedicarboxylic catalyst (Fe-NDC) for syngas conversion to olefin

**DOI:** 10.1038/s41598-025-09332-0

**Published:** 2025-07-22

**Authors:** Ahmed E. Rashed, Mohamed S. Nofal, Ahmed Abd El-Moneim

**Affiliations:** 1 Environmental Sciences Department, Faculty of Science, Alexandria University, Alexandria, 21511 Egypt; 2 Institute of Graduate Studies and Research, Alexandria University, Alexandria, Egypt; 3 Graphene Center of Excellence, Egypt-Japan University of Science and Technology, New Borg El Arab, 21934 Egypt; 4 Basic and Applied Science Institute, Egypt-Japan University of Science and Technology, New Borg El Arab, 21934 Egypt; 5 Physical Chemistry Department, National Research Centre, El-Dokki, Cairo, 12622 Egypt

**Keywords:** Thermally stable MOF, Syngas conversion, Iron catalyst, Olefin production, Catalyst synthesis, Heterogeneous catalysis, Chemical engineering, Organic-inorganic nanostructures

## Abstract

Olefins are the backbone of the petrochemical conversion industries, like polymers, plastic, lubricating oil, surfactants, and synthetic fuels. It is a wide but challenging process to customize. Metal–organic frameworks (MOFs) are highly regarded for their potential in Fischer–Tropsch synthesis (FTS), yet they often have inadequate thermal stability. This study demonstrated the remarkable potential of the Fe-NDC MOF. It maintains its initial structure until it reaches a temperature of 500 °C (Fe@C-500), which is efficient for syngas conversion to olefin. The Fe@C-500 catalyst exceeded a twofold increase in the ratio of olefin to paraffin compared to Fe@C-600 (2 vs. 0.8). The maintained structure of Fe@C-500 enhances the transport of reactants and restricts the hydrogenation of olefins. The Fe@C-500 catalyst showed ~ 50% and 27% selectivity to total olefin and light olefin, respectively, with a Fe-time yield (FTY) for light olefins of 180 mmol_CO_ g^−1^_Fe_ h^−1^. In contrast, Fe@C-600 exhibits a shift in product selectivity towards paraffin (~ 70%) at a lower FTY for light olefins of 130 mmol_CO_ g^−1^_Fe_ h^−1^. The performance of the Fe@C-500 catalyst is particularly intriguing and warrants further investigation. Retaining the porous structure of MOF-derived catalysts might greatly enhance olefin production.

**Supplementary Information:**

The online version contains supplementary material available at 10.1038/s41598-025-09332-0.

## Introduction

Considerable research effort has been devoted to developing sustainable alternative feedstocks for critical petrochemical products in response to rising concerns about the carbon footprint of fossil fuel consumption^1,2^. Designing optimized pathways for converting carbon emissions into valuable compounds and high-quality, sustainable hydrocarbon fuels is critical for establishing a carbon–neutral society^3^. Modern sustainable technologies, such as Power-to-X (PtX), biomass gasification to syngas, and emission-to-liquid (EtL) relying on Fischer–Tropsch Synthesis (FTS), are now cost-effective, large-scale options for fuel production, especially olefins^4,5^. Olefin supply and cost affect the global economy. Light olefins are the building blocks of essential materials such as polymers, plastics, and solvents^6^. Heavy olefins produce fuels such as gasoline by fluid catalytic cracking^7^. Biomass to liquid (BTL) and emissions to liquid (ETL) are receiving more attention because of the growing demand for clean and low-carbon energy and the enforcement of stricter environmental policies on the fuel industry^8,9^.

Olefins production relying on the FTS approach typically requires supported catalysts to foster a complex series of heterogeneous chemical reactions. The careful selection of catalyst and support contributes to keeping the Fischer–Tropsch reaction stable, active, and selective^10^. Carbon-supported iron nanoparticles are frequently studied for FTS activity due to the synergetic effect of iron’s high activity under broad process conditions and the mild interaction with the thermally stable carbon supports, ensuring better dispersion and reducibility^11,12^. Added to the abundance of cheap iron sources, various economic carbon materials have been investigated, such as biomass^13^, carbon fiber^8,14^, activated carbon^15,16^, graphene^17–19^, carbon nanotubes^20,21^, and more recently, Metal–Organic Frameworks (MOFs)^22–25^.

MOFs are exceptionally porous materials with a versatile crystalline structure composed of organic linkers and metal clusters^26^. Unlike common catalysts, MOFs avoid using post-synthetic treatments (e.g., impregnation) that decrease porosity and suppress active sites. MOF pyrolysis results in catalysts with metal dispersion at the atomic level in a carbon matrix^27,28^. While high metal loading normally limits dispersion and surface area (restricting at ~ 30 wt%), MOF-based catalysts overcome this barrier by achieving both high metal loading and well-dispersed small nanoparticles^29,30^. MOFs exhibit inferior thermal and chemical stability than typical inorganic porous materials, limiting their usage in thermocatalytic processes^31^. While certain MOF-based catalysts are quite resilient, their stability must be demonstrated experimentally. Additionally, the high cost of some organic linker precursors must be rationalized by the catalyst’s activity and product yield. Researchers make ongoing efforts to optimize these catalysts to increase the economic viability and efficiency of the process^32^. One attractive strategy is exposing catalysts to the pyrolysis process. Pyrolysis is a thermal treatment method where a material is exposed to elevated temperatures in an inert or reducing environment. Pyrolysis has shown potential in adjusting the active phase, surface area, and porosity, or incorporating promoters into the catalyst structure to enhance FTS-related challenges such as catalytic efficiency, product selectivity, and lifetime^33–35^.

Several studies applied the pyrolysis-mediated strategy to derive Fe nanoparticles on carbon support from Fe-MOF precursor with high loading and degree of dispersion^7,36–39^. For example, through the pyrolysis of Fe-MIL-88B and Fe-MIL-88B NH_2_, An et al.^37^ prepared active iron carbide catalysts with high catalytic activity. At various pyrolysis temperatures and in response to the addition of structural promoters, the Gascon group reported a series of catalysts fabricated from diverse MOFs, including Basolite F300, MIL-88A, MIL-127, MIL-68, MIL-101-NH_2_, and MIL-100, that exhibited distinct active iron carbide phases^34,36,40–42^. Their findings determined that the characteristics and structure of the initial MOFs, along with pyrolysis temperature, significantly impact the resulting catalyst in the context of catalytic efficiency and product selectivity.

Prior research on the pyrolysis of Fe-MOF for FTS aimed to fully break down the MOF structure to produce a carbon-supported catalyst with a high concentration of homogenously distributed iron. The pyrolysis temperature must be equal to or greater than the catalyst reduction and FTS reaction temperatures (up to 450 °C) to maintain the thermal stability of the catalyst throughout the process. The ideal temperature for Fe-MOF pyrolysis, based on prior studies spanning ranges from 400 to 900 °C, is 500 °C^34^. Maintaining the structural integrity of the MOF following pyrolysis at 500 °C without full collapse is challenging. The key is to increase the thermal stability of the MOF structure. Applying the MOF linker with multiple aromatic rings is expected to enhance the thermal stability of MOFs. The expanded aromatic system provides enhanced π-π Stacking, a stronger metal–ligand bond, and effective heat transfer, slowing thermal breakdown^43–45^. Moreover, a larger aromatic surface area limits water penetration into the framework and maintains metal–ligand bonds^46^. During FTS, it might help prevent catalyst deactivation by the water formed.

The 2,6-naphthalene dicarboxylic acid (NDC) is a linker used to synthesize Zr-NDC and Fe-NDC (MIL-142B) MOFs^47,48^. The NDC linker has two benzene rings in its structure, unlike conventional carboxylic acid linkers with one aromatic ring, such as benzene dicarboxylic (BDC) and tricarboxylic (BTC) acid. Zhang et al.^48^ reported the synthesis of Zr-NDC MOFs stable above 500 °C compared to Zr-BDC UiO-66(Zr) (400–500 °C)^43,45,49^. Higher Hydrophobicity for the NDC linker provides better resistance to hydrolysis than the BDC linker^46^. The prepared Fe-NDC catalyst shows superior thermal stability to other mono-benzene MOF peers, as confirmed by thermogravimetric analysis (TGA) conducted under inert and oxygen-rich environments. As far as we know, it is the first time using the partially pyrolyzed and thermally stable Fe-NDC MOF catalysts for the FTS process.

In brief, we demonstrate the solvothermal synthesis of Fe-NDC MOF, followed by pyrolysis at varying temperatures (500 °C and 600 °C) as considered by TGA. The catalysts were examined using thorough characterization methods to assess pyrolysis-induced changes. The catalysts were tested for FTS activity in a solar-powered fixed-bed reactor utilizing green hydrogen produced by electrolysis of solar desalinated water. The tests were conducted at a semi-industrial high gas hourly space velocity (GHSV) of 20,000 mL g^−1^cat h^−1^. In this study, we examine the pyrolysis temperature to tailor the properties of the resulting catalysts at a lower temperature while maintaining the original MOF structure intact. These catalysts are intended for use in a green FTS system to support sustainable and efficient hydrocarbon production.

## Methods

### Materials

Ferric Nitrate nonahydrate (Fe(NO_3_)_3_·9H_2_O) (≥ 98% purity) and n-octane (≥ 99% purity) were purchased from Fisher Scientific, UK, with laboratory reagent grade. 2,6-naphthalenedicarboxylic acid (99%), N,N-dimethylformamide (ReagentPlus®, ≥ 99%), and N-dodecane (ReagentPlus®, ≥ 99%) were provided by Sigma-Aldrich, USA. γ-Al_2_O_3_ (340 m^2^ g^−1^) was acquired from BASF. An on-demand hydrogen generating system (H-Genie®) at 100 bar pressure produced grade 4, 99.99% hydrogen gas from pure water. Post-electrolyzer purification system yields grade 5, 99.999% hydrogen gas. All other employed gases supplied or imported by Air Supply Co., Egypt, with grade 5, 99.999%.

### Catalysts synthesis

*Synthesis of Fe-NDC (MIL-142B) MOF:* The Fe-NDC MOF was prepared according to Ibrahim et al.^47^ using the solvothermal method by dissolving 0.692 mmol (279.6 mg) of iron nitrate nonahydrate (Fe(NO_3_)_3_.9H_2_O) and 0.692 mmol (149.7 mg) of 2,6-naphthalenedicarboxylic acid in 30 mL of N,N-dimethylformamide (DMF). Subsequently, the solution was transferred into a 60-mL Teflon autoclave and heated in an oven at a temperature of 100 °C for 24 h. The resultant yellow mixture underwent centrifugation and was rinsed twice with fresh DMF to eliminate any remaining unreacted components. Eventually, the developed MOF was dried in a vacuum oven at 100 °C to eliminate residual solvent.

*Preparation of Fe-NDC derived catalyst by pyrolysis:* The dehydrated MOF was milled and then exposed to pyrolysis in a nitrogen stream for 4 h at two different temperatures, namely 500 and 600 °C. The temperature was held constant at 60 °C for 1 h, after which it was gradually raised to 500 or 600 °C at a rate of 5 °C per minute. Finally, the produced catalysts Fe@C-500 and Fe@C-600 were passivated by gradually exposing them to a continuous flow of 1% O_2_/Ar gas mixture to avoid combustion upon exposure to the atmosphere.

*Preparation of* Fe@Al_2_O_3_ *catalyst:* An iron reference catalyst supported on Alumina (with a Fe mass fraction of 30%) was synthesized using the incipient wetness impregnation technique (IWI) according to previous work^7^. To guarantee complete incorporation within the pore volume of alumina, a solution of Fe(NO_3_)_3_·9H_2_O (15.192 g, 37.6 mmol) was introduced onto the one gram alumina until it was thoroughly wetted. After drying, it was subjected to calcination in air at a temperature of 500 °C for 2 h, with a heating rate of 10 °C per minute.

### Catalyst characterization

The X-ray diffraction (XRD) patterns were generated using a Shimadzu XRD-6100 instrument, utilizing Cu-Kα radiation within the 10–80° range to identify the present phases. The determination of particle size was performed using the Debye–Scherrer equation using MDI Jade software (v. 6). The chemical characteristics change before and after pyrolysis were investigated using Fourier-transform infrared spectroscopy (FTIR) spectra produced using the Bruker Vertex 70 instrument.

The iron content of the MOF-based catalyst was assessed using thermogravimetric analysis (TGA) conducted under air and atomic absorption spectroscopy (AAS). The TGA-50 instrument from Shimadzu was used, with a temperature range of 25–800 °C and a heating rate of 10 °C per minute. Iron loadings were estimated via TGA using formulae derived from Oar-Arteta et al.^41^, assuming Fe_2_O_3_ is the main oxidized phase. The chemical and electronic characteristics of the surface were investigated via X-ray photoelectron spectroscopy (XPS) employing K-ALPHA (Thermo Fisher Scientific, USA) equipped with monochromatic X-ray Al Kα radiation ranging from −10 to 1350 eV, a spot size of 400 µm and a pass pressure of 10–9 mbar, pass energy of 200 eV for the entire spectrum, and 50 eV for the narrow spectrum.

Scanning and transmission electron microscopy (SEM and TEM–EDX) are two techniques used for imaging and analyzing materials at the microscopic level. JEOL JSM-6010LV and JEOL JEM-2100F microscopes were used to obtain images to examine the morphological structure, particle size distribution, and dispersion. The N_2_ adsorption/desorption isotherms at 77 K were measured by BEL Japan (Belsorp II mini) and analyzed using the Brunauer–Emmett–Teller (BET) and BJH t-plot techniques to obtain the BET surface area, mean pore diameter, and total pore volume. Before measurement, the samples were degassed at 150 °C for a whole night. The H_2_-temperature-programmed reduction (H_2_-TPR) data were collected using a MICROTRAC BELCAT II catalyst analyzer fitted with a thermal conductivity detector. This data was used to determine the sequence and extent of reduction. Data was collected from ambient temperature to 900 °C, with a heating rate of 10 °C per minute, while a stream of 30 mL per minute containing 5% H_2_/Ar was used. Chemisorption measurements for CO and H_2_, specifically temperature-programmed desorption (TPD), were conducted using the MICROTRAC BELCAT II catalyst analyzer.

### Catalytic performance evaluation

**Fig. 1:**
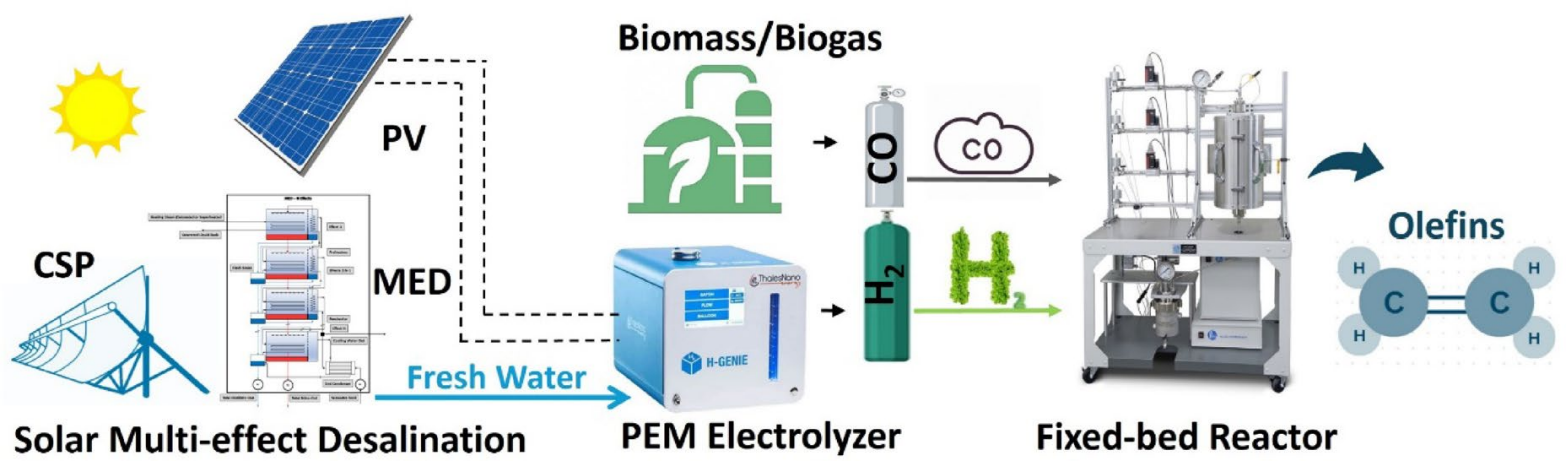
Process scheme of the Power-to-X system.

Figure 1 demonstrates the components of the applied Power-to-X system. Green hydrogen was generated from a Proton Exchange Membrane (PEM) water electrolyzer. Fresh water was supplied by a multi-effect desalination (MED) system running on solar energy from concentrated solar power (CSP). A photovoltaic (PV) system powers the electrolyzer. The choice of the fixed bed reactor material was stainless steel (316 grade), primarily based on its cost-effectiveness and high degree of corrosion resistance compared to other materials^50–52^. The reactor furnace’s temperature was calibrated based on earlier research^53^.

For each trial, the fixed bed reactor was filled with the catalyst (0.5 g) and an equivalent amount of SiC. A control experiment using identical reaction conditions revealed that the reactor, glass beads, glass wool, and SiC were devoid of catalytic activity. The catalyst was reduced using a flow rate of 50 ml per minute of hydrogen gas for 4 h at a temperature of 400 °C, with a heating rate of 10 °C per minute. Subsequently, the catalyst was cooled down to a temperature of 180 °C. Upon reaching the target temperature of 340 °C, syngas was introduced at a Gas Hourly Space Velocity (GHSV) of 20,000 mL g^−1^cat h^−1^, under a pressure of 20 bar, and with an H_2_/CO ratio of 1. A cryogenic trap containing n-octane (2 g) was used to capture a portion of liquid hydrocarbon at a temperature of 3 °C.

Permanent gas composition was monitored using a Shimadzu-GC-2014 GC/TCD instrument with a 3 m Shincarbon Restek Column. The three gases’ molar concentrations were verified using an external standard. He used a carrier gas with a flow of 20 ml min^−1^, heating the injection ports to 100 °C, the column to 120 °C, and the TCD detector to 180 °C. A Shimadzu-GC-2014 GC/FID system with a Rt-Alumina BOND/Na_2_SO_4_ column (30 m, 0.32 mm ID, 5 µm film) was used to identify the light hydrocarbon fraction (C_1_–C_6_). For light hydrocarbon calibration, a petroleum company supplied C_1_–C_6_ reference gas into the GC/FID system. After 6 min at 40 °C, the FID oven was heated to 100 °C (15 °C min^−1^) for 37 min. Samples were injected at a 150:1 split ratio with 40 cm s^−1^ helium linear velocity.

Using a GC/FID system (SRI-8610C-GC), the liquid fraction was fed onto an MXT-1 Restek column (60 m, 0.53 mm ID, 5 m film) with an internal standard of N-dodecane. After 3 min at 35 °C, the column’s oven temperature was increased to 240 °C (5 °C min^−1^), then maintained at 300 °C (10 °C min^−1^) for 60 min at 5 ml min^−1^ He. The olefin yield and selectivity of liquid samples were measured following sulfuric acid treatment. The catalyst performance was determined using previously reported calculations^54^. The average catalytic activity in moles for feed carbon (CO) and product carbon (residual CO, CO_2_, and hydrocarbons) was used to estimate the carbon balance.

## Results and discussion

### Catalyst characterization

**Fig. 2:**
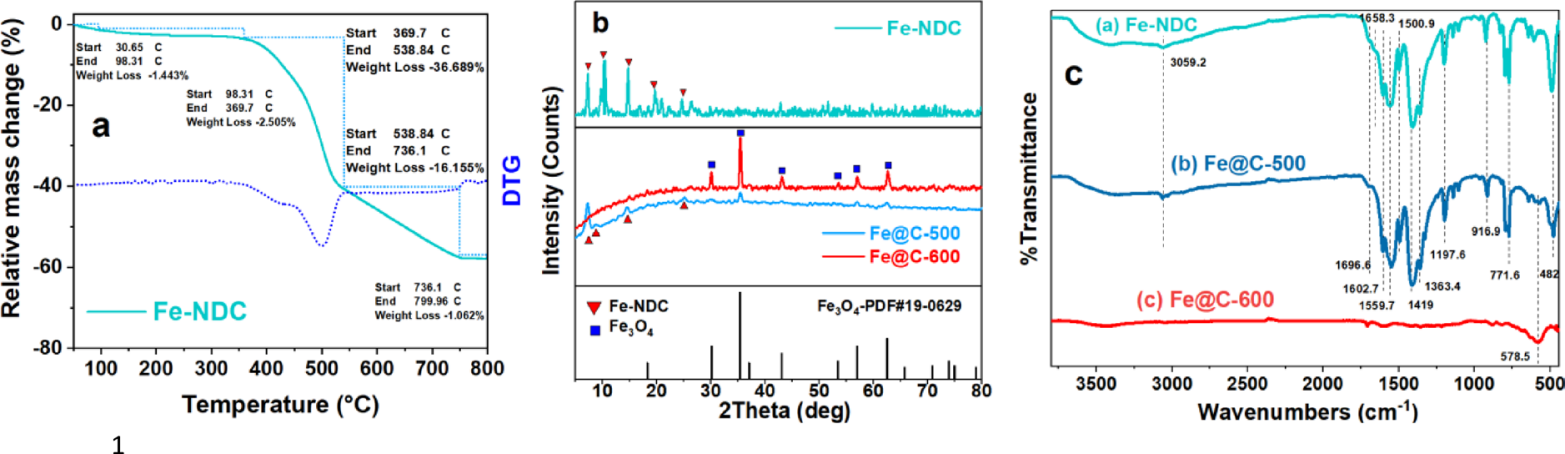
TGA and DTG of Fe-NDC (**a**), XRD patterns (**b**), and FTIR spectra (**c**) of Fe-NDC MOF and derived catalysts. MOF under nitrogen flow.

TGA profiles were produced for fresh Fe-NDC MOF under an inert atmosphere (Nitrogen) to investigate thermal stability and determine the suitable pyrolysis temperature. Figure 2a shows that weight loss increased as temperature rose throughout two successive stages. The first weight loss seen between room temperature and 369 °C (about 4% weight loss of Fe-NDC) is likely due to the elimination of moisture and DMF solvent molecules from the MOF’s porosity. A significant breakdown occurred between 369 and 538 °C, resulting in a weight loss of roughly 36.7 wt% of the original mass. This may be attributed to the heat decomposition of the structurally coordinated 2,6-NDC ligands. Another weight loss occurred between 538 and 736 °C, resulting in a mass loss of 16.2 wt%. The loss is likely caused by the breakdown of the iron metal clusters from the MOF framework^47^. The findings proved that Fe-NDC-MOF is more thermally stable than conventional Fe-MOFs previously reported ^7,38^. The respective weight losses resulting from the two stages are 36.7% and 16.2%. Therefore, a temperature of 500 °C was anticipated to cause partial pyrolysis of the MOF. The pyrolyzed MOF at 600 °C likely resulted in the complete disintegration of the MOF structure.

Figure 2b depicts the XRD patterns of Fe-NDC MOF and active catalysts. The presence of distinct diffraction peaks (2θ = 7.2°, 9.7°, 10.2°, 10.5°, and 14.7°) provides confirmation that the synthesized MOFs possess well-defined crystalline structures, matching published research^47,55^. Unlike Fe@C-500, the XRD pattern of Fe@C-600 is free of MOF characteristic diffraction peaks, confirming the collapse of the MOF framework, see Fig. 2b. The XRD patterns reveal that magnetite (Fe_3_O_4_) is the predominant iron phase, exhibiting a fcc structure as per the JCPDS file, No. 19-0629^41^. Fe@C-500 pyrolyzed at a lower temperature shows pronounced peaks at 7.2°, 14.7°, and 25.1°, confirming the residual structure of the fresh MOF structure. Reduced magnetite peaks suggest a smaller particle size of Fe@C-500.

As illustrated in Supplementary Fig. S1, the differential thermogravimetric (DTG) and TGA profiles of fresh and pyrolyzed Fe-NDC were generated in the air. The first weight reduction in the TGA curves of the parent MOF, occurring before 300 °C, is likely due to the volatilization of solvents and water. Fe-NDC displayed a two-step substantial weight loss, with the highest rate of MOF breakdown seen at 355 °C and 435 °C. The first loss begins at 300 °C and concludes at 490 °C. The second loss occurs between 490 and 690 °C. The respective weight losses resulting from the two stages are 54% and 15%. Therefore, a temperature of 500 °C was anticipated to cause partial pyrolysis of the MOF. The pyrolyzed MOF at 600 °C likely resulted in the complete disintegration of the MOF structure. The XRD results indicate that the material was not completely destroyed at 500 °C since specific peaks typical of MOF were still present, Fig. 2b. TGA was used to determine the Fe loading pre- and post-pyrolysis. The remaining mass was projected to consist only of Fe_2_O_3_. The Fe loadings derived from TGA and AAS had comparable percentages, as seen in Table 1.

**Table 1:** Fe particle size from XRD and TEM, Fe loading obtained by TGA and AAS, and XPS surface atomic percentage of MOF and derived catalysts.

Sample	Fe wt%^a^		d_Fe_ (nm)^b^		XPS, at%^c^			
	TGA	AAS	TEM	XRD	C_1s_	O_1s_	Fe_2p_	N_1s_
Fe-NDC	9.4	10.5	–	–	–	–	–	–
Fe@C-500	25.8	24.0	27.3	22.4	74.77	20.92	3.68	0.63
Fe@C-600	27.4	29.3	38.1	30.5	89.47	8.1	1.96	0.47

^a^ Fe weight percent, ^b^ Fe average particle size, ^c^ atomic surface distribution.

Other characterization techniques were needed to evaluate the different properties of the MOF and the produced catalysts. The IR spectrum of Fe-NDC in Fig. 2c is similar to those in earlier studies^47,55^. The FTIR data suggest that iron ions are fully coordinated with the NDC linker. The carboxylic acid vibrations related to NDC, − OH (3059–2500 cm^−1^), and C = O stretching (1696 and 1300 cm^−1^) were either minimal or undetected. The weak bands at 1658 and 1696 cm^−1^ correspond to traces of DMF and unbound NDC acid within the pores, respectively. On the other hand, strong coordination bands associated with the vibrations of C = O, –COO − , and out-of-plane − CH (1602, 1559, 1500, 1419, 1363, and 771 cm^−1^) of the aromatic NDC were detected. In addition, the two peaks at 1197 and 916 cm^−1^ correspond to the in-plane − CH vibrations. Furthermore, the 482 cm^−1^ band, characteristic of Fe–O, is shown in Fig. 2c. The findings confirm the effective coordination of Fe^3+^ ions with NDC to form Fe-NDC crystals. Following the pyrolysis of the produced Fe-NDC at 600 °C, a significant decrease in coordination peaks indicates successful pyrolysis. The strong band observed at 578 cm^−1^ and the less pronounced one at 482 cm^−1^ resemble the characteristics of magnetite as previously documented^56^, in accordance with XRD findings. The Fe-NDC MOF shows partial collapse at a lower pyrolysis temperature of 500 °C, as confirmed by the presence of most of the IR peaks of the original MOF. Strong Fe–O vibrations from the MOF structure are seen at 482 cm^−1^, along with a weak magnetite band at 578 cm^−1^.

**Fig. 3:**
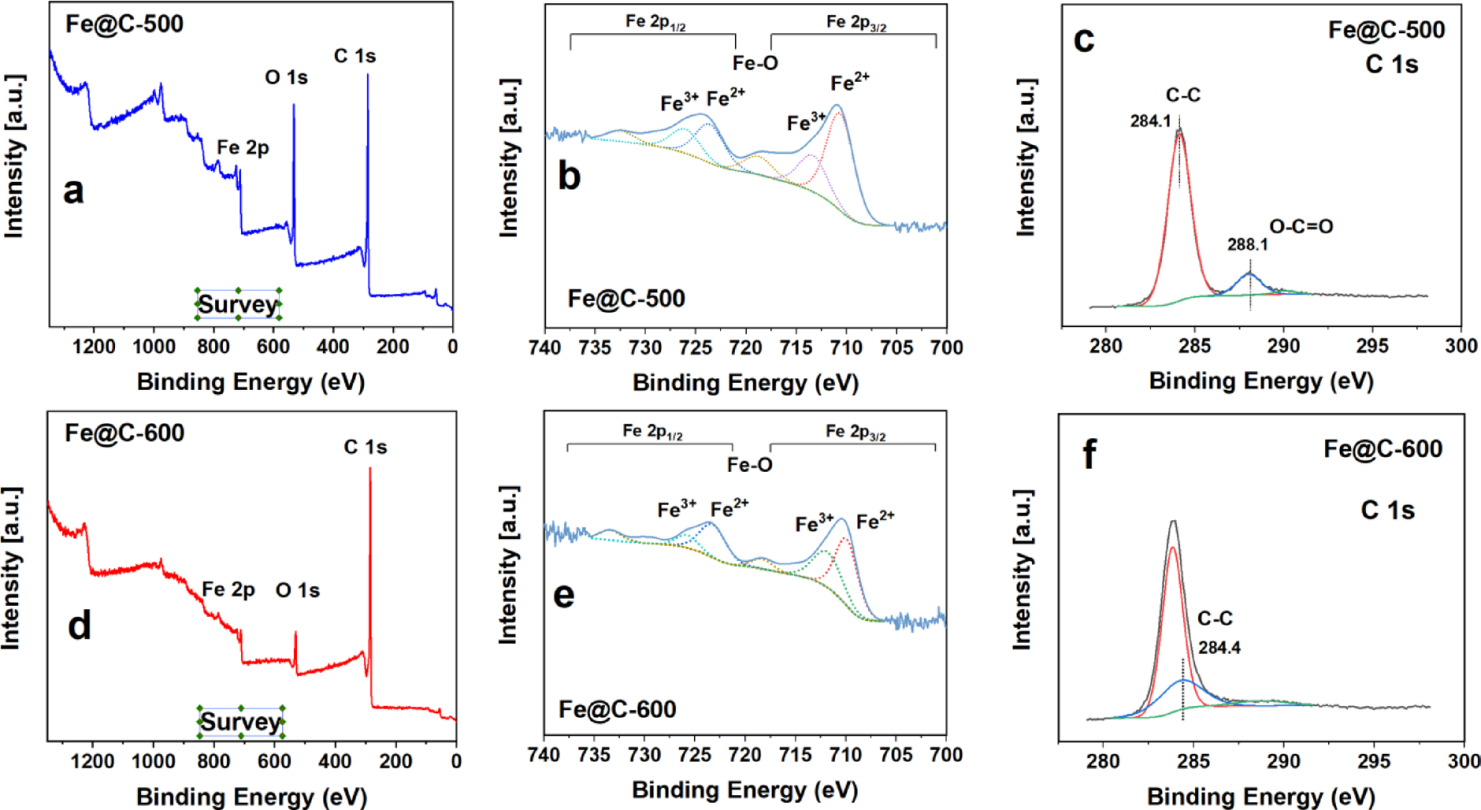
XPS spectra of (**a**–**c**) Fe@C-500 and (**d**–**f**) Fe@C-600.

As XRD findings provide information on the bulk phase only, XPS of Fe2p, O1s, and C1s was conducted to analyze the chemical structure and electronic characteristics at the surface, Fig. 3. Fe2p core level spectra of Fe@C-500 and Fe@C-600 are shown in Fig. 3b and e. The leading bands at 724.5 eV and 710.8 eV suggest the existence of Fe_3_O_4_. The resolved peaks at 710.8 and 713.5 eV (Fe 2p_3/2_) and 723.8 and 726.2 eV (Fe 2p_1/2_) are attributed to Fe(II) and Fe(III) correspondingly^36,57^. A satellite peak at 719 eV and Fe 2p_1/2_ shake-up of about 733.4 eV were also identified^58^. No metallic iron is apparent in the Fe 2p_3/2_ and Fe 2p_1/2_ spectra^36^, consistent with X-ray diffraction patterns.

The amorphous C–C configurations are the main feature in the C1s spectra of the catalysts, appearing at binding energies between 284.1 and 284.4 eV^59^, refer to Fig. 3c and f. This characteristic promotes the even distribution of iron oxide, facilitating the creation of active surface iron carbide species when exposed to syngas^60^. At elevated heat treatment, the absence of the O–C = O peak (288.1 eV) indicates the elimination of oxygen species due to the breakdown of the coordinated NDC linker and its transformation to carbon species^61^, as verified by XRD and FTIR.

XPS spectra of the O1s core level for Fe@C-500 and Fe@C-600 are shown in Supplementary Fig. S2. Fe@C-500 exhibits two distinct surface oxygen species: the significant bands at 529.8–531.2 eV indicate the presence of Fe oxide phases (specifically FeO-MOF cluster and Fe_3_O_4_), whereas the bands with higher binding energies at around 532.2 eV are attributed to the surface oxygen C = O group of the preserved structure of the Fe-NDC MOF, as confirmed by XRD^36^. The O1s XPS spectra of Fe@C-600 exhibit a lower representation of surface oxygen groups, as presented in the survey Fig. 3a and c. The lower heat treatment effectively maintains a greater O/Fe ratio and the relative quantity of oxygen groups on the surface of the porous carbon. On Fe@C-500, the relative surface percentage of oxygen is 20.92%, but on Fe@C-600, it reduces to 8.1%.

XPS analysis indicates that the concentration of Fe atoms on the outer surface of Fe@C-500 is double that of Fe@C-600, demonstrating that a higher pyrolysis temperature results in increased encapsulation of the Fe phase inside the carbon matrix. The Fe@C-500 treatment maintained the MOF structure and Fe–O clusters, increasing Fe and O surface atomic%. The increased heat treatment (Fe@C-600) led to a more significant conversion of –OH and –COOH groups into carbon-based support, decreasing the surface atomic% of oxygen. The carbon matrix covered the Fe nanoparticles, leading to a decrease in Fe and an increase in C percentage on the surface. Refer to Table 1 for XPS atomic percentages.

**Fig. 4:**
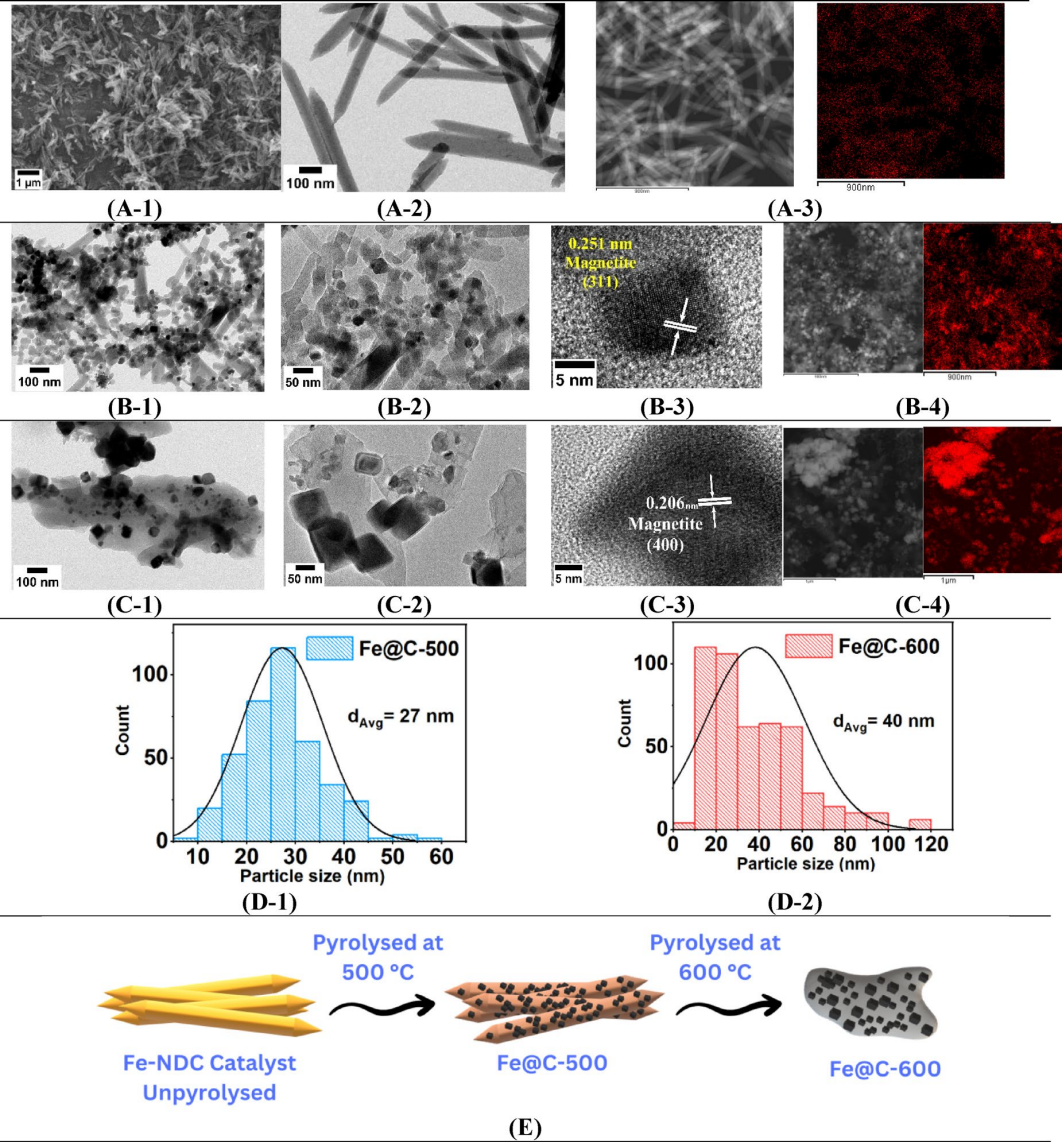
SEM, TEM, HRTEM, STEM, and iron elemental mapping (red) images of (**A**) Fe-NDC, (**B**) Fe@C-500, and (**C**) Fe@C-600. (**D**) PSD of derived catalysts. (**E**) Schematic of the structural change at different pyrolysis temperatures.

Figure 4 displays SEM and TEM images showing consistent crystalline hexagonal rods of fresh Fe-NDC MOF (Fig. 4(A-1 and A-2)). Following pyrolysis, the TEM images show evenly distributed Fe nanoparticles on the hexagonal rods for Fe@C-500 (Fig. 4(B-1 and B-2)) with sizes between 10 and 50 nm and an average size of 27 nm, as determined by particle size distribution (PSD) analysis (Fig. 4(D-1)). Particles ranging in size from 10 to 120 nm, with an average size of around 40 nm, Fig. 4(D-2), were identified for Fe@C-600 scattered on a carbon matrix, Fig. 4(C-1 and C-2). Elemental mapping of pyrolyzed Fe-NDC indicates that Fe@C-500 exhibits superior dispersion of iron with high intensity in the carbon matrix compared to Fe@C-600, as shown in Fig. 4(B-4 and C-4). In agreement with the XRD data, the HR-TEM images (Fig. 4(B-3 and C-3)) of pyrolyzed MOF reveal the interplanar spacings of 0.251 nm and 0.206 nm, which match the (311) and (400) lattice planes of the magnetite fcc structure. Based on the above results, the structural change of Fe-NDC MOF at different pyrolysis stages is suggested in Fig. 4E. After 500 °C pyrolysis, the parent Fe-NDC MOF’s retained hexagonal rods contain well-dispersed Fe nanoparticles. Following the collapse of the hexagonal rod structure at 600 °C, larger Fe nanoparticles were obtained and distributed in a carbon matrix.

**Fig. 5:**
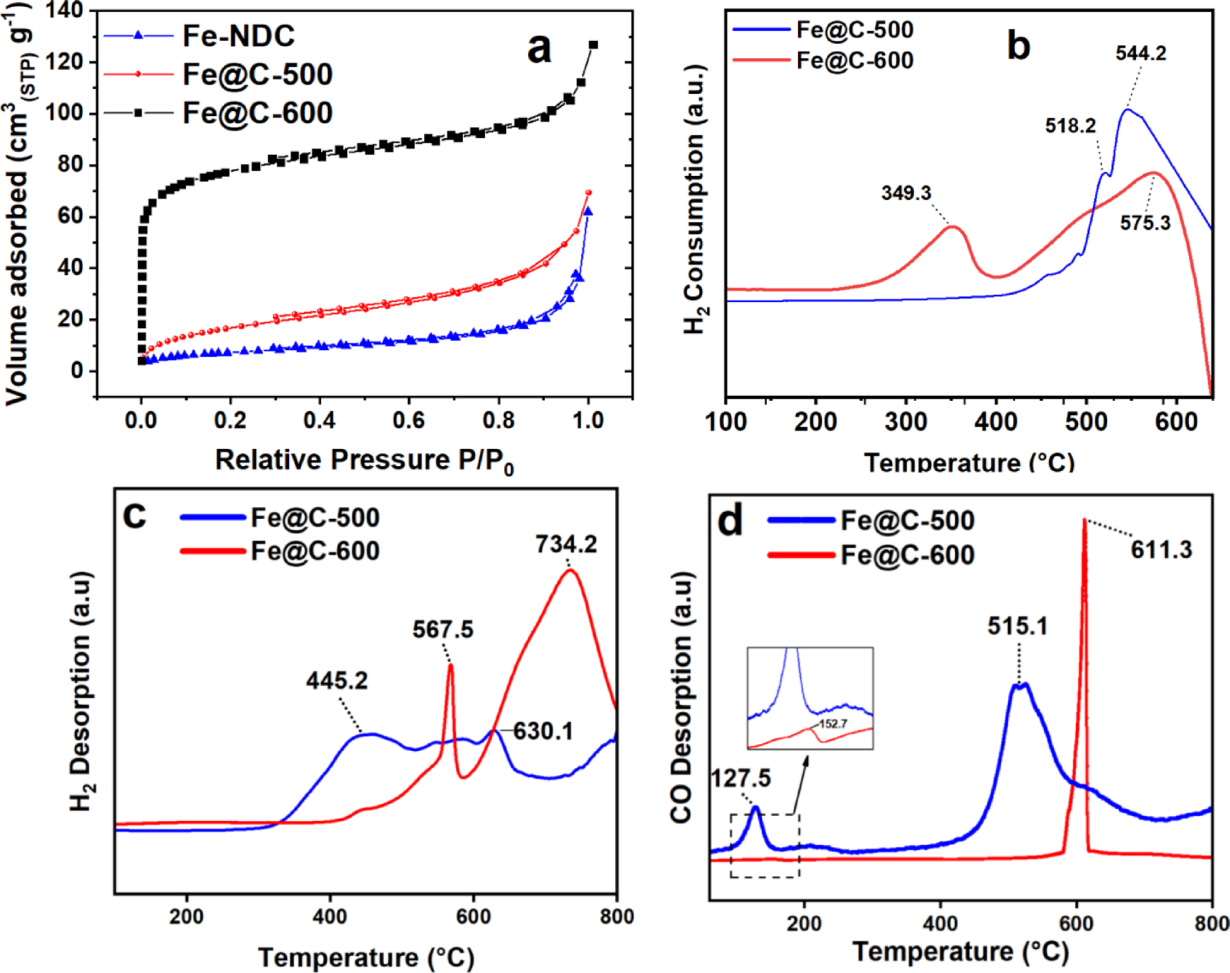
(**a**) Nitrogen adsorption isotherms of Fe-NDC and derived catalysts. (**b**) H_2_-TPR. (**c**) H_2_ and (**d**) CO chemisorption of derived catalysts.

BET measurements were used to analyze the surface characteristics of the catalyst by comparing the Fe-NDC sample before and after pyrolysis. The BET Analysis indicates that Fe-NDC MOF has a comparatively low BET surface area of 25.9 m^2^ g^−1^ and a non-microporous adsorption behavior in contrast to the reported literature^55^; see Table 2 and Fig. 5a. The decreased surface area and lack of microporous isotherm might originate from the tunnel-shaped pores^55^ in the Fe-NDC structure being obstructed by remnants of DMF solvent or free NDC acid, evidenced by the FTIR spectra. Following pyrolysis at 500 °C, the adsorption–desorption isotherm exhibited a slight increase in surface area (62.1 m^2^ g^−1^) and total pore volume with respect to the original MOF. Increasing the pyrolysis temperature from 500 to 600 °C resulted in a higher amount of N_2_ adsorbed, suggesting an expansion of the porous structure due to the collapse of the MOF caused by carbon gasification.

**Table 2:** Textural properties, TPR, and chemisorption of Fe-NDC and derived catalysts.

Sample	S (m^2^ g^−1^)		V (cm^3^ g^−1^)		D_pore_^d^ (nm)	Chemisorption (mmol g^−1^)		
	S_BET_^a^	S_meso_^b^	V_total_^c^	V_meso_^b^		H_2_^e^ Consumed	H_2_^f^ Uptake	CO^g^ Uptake
Fe-NDC	25.9	21.8	0.074	0.063	11.54	–	–	–
Fe@C-500	62.1	51.13	0.01	0.092	6.29	7.251	3.621	0.573
Fe@C-600	282	55.92	0.18	0.082	2.5	1.99	6.613	2.197

^a^ BET surface area, ^b^ BJH mesoporous surface area and volume, ^c^ Total pore volume at (p/p_0_ = 0.990), ^d^ Average pore size, ^e^ H_2_ consumption from TPR, ^f^ H_2_ uptake, and ^g^ CO uptake from chemisorption.

Figure 5a shows that Fe@C-600 combines type-I and type-IV isotherms. Type-I microporosity originates from the parent MOF following the breakdown of free NDC acid, whereas mesoporosity results from the liberation of carbon due to MOF failure^8^. The BET surface area increases from 62.1 to 282 m^2^ g^−1^ after the temperature rises from 500 to 600 °C. The total volume increases, as well, from 0.01 to 0.18 cm^3^ g^−1^. The larger surface area and mesoporosity of the carbon support may aid in the diffusion of gases, leading to improved CO conversion^8^.

The TPR profiles of Fe@C-500 and Fe@C-600 in Fig. 5b indicate hydrogen utilization of 7.25 and 1.99 mmol g^−1^, highlighting the greater surface exposure of Fe nanoparticles in Fe@C-500, as validated by elemental mapping and Fe surface atomic% from XPS in Table 1. Fe@C-500 consumed more hydrogen due to the reduced amount of oxygen surface groups. Figure 5b illustrates two significant reduction stages for both catalysts. In the first two peaks, Fe_3_O_4_ is reduced to FeO and FeO is reduced to Fe, respectively. This aligns well with the reduction characteristics of iron oxides^28,37^. The higher heat treatment shifted the first reduction peaks to lower values (349 °C) for the Fe@C-600 catalyst, compared to Fe@C-500 (518 °C), as the Fe–O clusters in the maintained MOF structure of Fe@C-500 need more energy to be reduced. Beyond 600 °C, negative peaks in both catalysts originate from removing –OH and –COOH groups remaining in the pyrolyzed MOF.

The catalyst was evaluated for H_2_ and CO chemisorption to determine its ability to adsorb the supplied gases. The olefin selectivity is anticipated to be influenced by the variation of CO and H_2_ bindings^58^. Figure 5c demonstrates both weak and strong H_2_ adsorption in the H_2_ desorption curve. Fe@C-500 exhibits reduced hydrogen affinity at lower desorption temperatures (455 °C and 630 °C) than Fe@C-600 (567 °C and 734 °C). Fe@C-600 shows double the hydrogen uptake in relation to Fe@C-500, as seen in Table 2. The data suggest that a catalyst subjected to lower heat treatment exhibits greater olefin selectivity due to reduced availability of hydrogen for olefin hydrogenation.

The CO desorption curve in the Fig. 5d indicates that Fe@C-600 exhibits a small CO desorption peak at 152 °C, which is attributed to its limited adsorption strength. An enhanced and more distinct CO desorption peak at 611 °C was observed, suggesting the liberation of strongly adsorbed CO, as shown in Fig. 5d. Fe@C-500 exhibits a noticeable weak-desorption peak at 127 °C and a less pronounced strong-desorption peak at 515 °C. Desorbed CO is greater for Fe@C-600 compared to Fe@C-500, as indicated in Fig. 5c and d and Table 2. The findings suggest that Fe@C-600 exhibits more catalytic activity for CO conversion because of its higher CO uptake and higher desorption temperatures.

### Catalytic activity results

#### FTS performance evaluation for prepared catalysts

The high thermal stability of the prepared Fe-NDC MOF, as shown by TGA results, provides the opportunity to pyrolyze the MOF partially. This could be done at a temperature (500 °C) higher than the FTS reaction conditions while retaining a large share of the MOF structure without a complete collapse of MOF driven by pyrolysis heat. Therefore, the FTS performance was compared between the prepared partially and fully pyrolyzed catalysts (Fe@C-500 and Fe@C-600). The catalytic performance of the catalysts and Fe/Al_2_O_3_ as a control catalyst was evaluated in the Fischer–Tropsch synthesis process under specific reaction conditions: T = 340 °C, P = 20 bar, GHSV = 20,000 mL g^−1^_cat_ h^−1^, and H_2_/CO ratio of 1. Table 3 summarizes the FTS CO conversion, Fe-time yield (FTY), chain growth probability (α), carbon balance, carbon selectivity/yield of various product ranges, and the O/P ratio values after 17 h of operation.

**Table 3:** FTS performance of MOF-derived and reference catalysts.

	Fe@C-500			Fe@C-600	Fe/Al_2_O_3_
GHSV (mL g^−1^_cat_ h^−1^)	4200	4200	20,000	20,000	20,000
P (bar)	20	20	20	20	20
T (°C)	300	340	340	340	340
X_CO_ %	68.56	71.86	66.08	89.74	69.5
Selectivity					
CO_2_	42.38	40.38	44.81	44.29	50.4
CH_4_	13.45	17.02	23.32	30.18	42.3
C_2_–C_4_	35.09	37.92	41.37	40.53	50.1
C_5+_	51.45	45.05	35.30	29.27	7.6
C_5_–C_12_	47.34	44.12	33.49	28.18	7.5
C_13+_	4.11	0.93	1.80	1.09	0.10
C_2_–C_4_ olefin	23.41	27.19	27.15	18.25	19.3
C_5+_ olefin	35.21	29.21	20.88	14.89	2.1
Total olefin	58.62	56.40	48.04	33.14	21.4
C_2_–C_4_ iso/paraffin	11.67	10.73	13.92	22.27	30.8
C_5+_ iso/paraffin	16.24	15.84	14.41	14.38	5.5
Paraffin	36.36	37.82	46.66	60.83	75.5
Isoparaffin	5.01	5.78	5.28	6.01	3.1
Total iso/paraffin	41.37	43.60	51.95	66.85	78.6
Olefin/paraffin					
O/P	1.41	1.29	0.92	0.49	0.27
O/P (C_2_–C_4_)	2.00	2.53	1.95	0.81	0.62
O/P (C_5+_)	2.16	1.84	1.44	1.03	0.38
Yield					
C_5_–C_12_	18.7	18.90	12.21	14.08	2.58
Total Olefin	23.16	24.16	17.52	16.56	7.37
Olefin (C_2_–C_4_)	9.25	11.64	9.90	9.12	6.65
Olefin (C_5+_)	13.91	12.51	7.61	7.44	0.721.41
Other					
α	0.65	0.64	0.57	0.56	0.39
C-balance	103.22	102.18	104.44	97.66	98
FTY (mmol_CO_g^−1^_Fe_ h^−1^)	164.03	177.71	660.8	709.7	257.8
FTY _C2–C4_ (mmol_CO_g^-1^_Fe_ h^−1^)	44.55	48.27	179.5	129.6	49.8
TOS	17	17	17	17	16

**Fig. 6:**
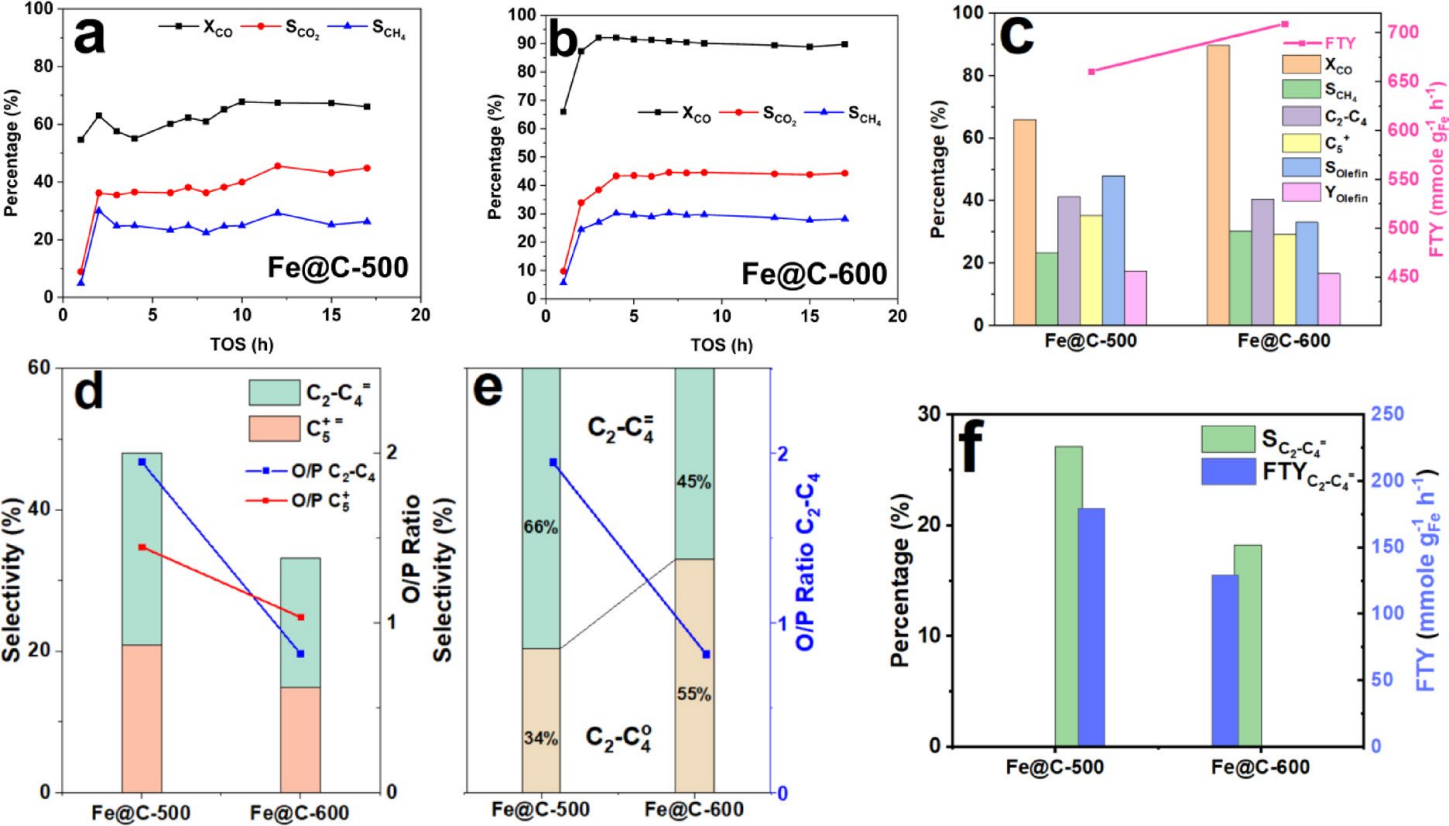
FTS activity of (**a**) Fe@C-500, (**b**) Fe@C-600 as a function of TOS, (**c**) Hydrocarbon distribution and FTY, and (**d**–**f**) olefin product distribution, at P = 20 bar, GHSV = 20,000 mL g^−1^_cat_ h^−1^, and H_2_/CO ratio of 1.

Figure 6a and b illustrates the changes in X_CO_ and selectivity of carbon dioxide (${\text{S}}_{{\text{CO}}_{2}}$) and methane $({\text{S}}_{{\text{CH}}_{4}})$ with TOS for Fe@C-500 and Fe@C-600 catalysts. Fe@C-600 has a higher X_CO_ (89.7%) than Fe@C-500 (66.1%), but with comparable ${\text{S}}_{{\text{CO}}_{2}}$ (44%) and FTY values (660.8 vs. 709.7 mmol_CO_g^−1^_Fe_ h^−1^). Figure 6c demonstrates the hydrocarbon selectivity distribution of $({\text{S}}_{{\text{CH}}_{4}})$, C_2_–C_4_ selectivity ${(\text{S}}_{{\text{C}}_{2}-{\text{C}}_{4}})$, ${\text{S}}_{{\text{C}}_{5+}}$, and FTY for Fe@C-500 and Fe@C-600 catalysts. Fe@C-600 shows higher hydrocarbon methane selectivity, whereas ${\text{S}}_{{\text{C}}_{2}-{\text{C}}_{4}}$ remains unchanged and ${\text{S}}_{{\text{C}}_{5+}}$ is moderately increased for Fe@C-500 at the same α values (0.56–0.57). Interestingly, Fe@C-500 and Fe@C-600 catalysts produce the same amount of olefin (olefin yield of 17.5% vs. 16.5%), even though Fe@C-500 has lower X_CO_ (66.1 vs. 89.7%) and greater olefin selectivity (48% vs. 33%).

Figure 6d–f shows the olefin product distribution of Fe@C-500 and Fe@C-600 catalysts. Figure 6d indicates that the Fe@C-500 catalyst has larger olefin selectivity and enhanced O/P. The overall O/P ratio of Fe@C-500 (0.92) is approximately twofold higher than Fe@C-600 (0.49), see supplementary Fig. S3b and Table 3. For Fe@C-500, the light olefin selectivity ${(\text{S}}_{{\text{C}}_{2}-{{\text{C}}_{4}}^{=}})$ = 27.2%, whereas the O/P of C_2_-C_4_ approaches 2 including 66% olefins of the C_2_-C_4_ fraction, Fig. 6e, and heavy olefins (O/P (C_5+_)) of 1.44, Fig. 6d. On the other hand, Fe@C-600 shows ${\text{S}}_{{\text{C}}_{2}-{{\text{C}}_{4}}^{=}}$ of 18.3%, O/P of C_2_–C_4_ of 0.8, which is less than half of the value of Fe@C-500, with only 45% of olefins in the C_2_–C_4_ fraction. In addition, a lower O/P (C_5+_) of 1.03 is observed for Fe@C-600. Fe@C-500 outperforms Fe@C-600, showing larger FTY for light olefins, Fig. 6f, ${(\text{FTY}}_{{\text{C}}_{2}-{{\text{C}}_{4}}^{=}})$ of 179.5 mmol_CO_ g^−1^_Fe_ h^−1^ compared to 129.6 mmol_CO_g^−1^_Fe_ h^−1^. It can be concluded that the O/P of C_2_–C_4_ and C_5+_ increase as the pyrolysis temperature of Fe-NDC decreases. Unlike the trend of olefin selectivity, the total olefin yield of C_2_-C_4_ and C_5+_ is relatively constant for both catalysts, as presented in supplementary Fig. S3b–d.

**Fig. 7:**
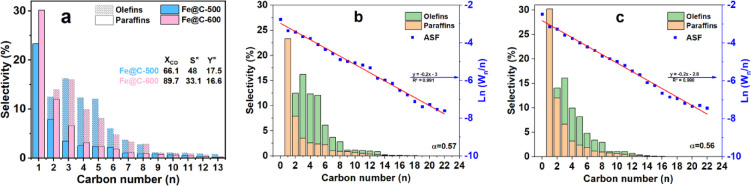
(**a**) Carbon number product distribution and ASF distribution of (**b**) Fe@C-500 and (**c**) Fe@C-600 at T = 340 °C, P = 20 bar, GHSV of 20,000 mL g^−1^_cat_ h^−1^, and H_2_/CO of 1.

Figure 7a summarizes the FTS activity and product distribution after 17 h TOS for Fe@C-500 and Fe@C-600 catalysts. Furthermore, Fig. 7b and c show the Anderson–Shulz–Flory (ASF) plot of the prepared catalysts. The catalysts’ product spectrum adheres strictly to the Anderson-Schulz-Flory distribution, with the chain growth probability marginally shifting from 0.56 (for Fe@C-600) to 0.57 (for Fe@C-500). Both catalysts show high catalytic activity and olefin yield. However, the Fe@C-500 catalyst shows relatively lower carbon monoxide conversion while having high olefin/paraffin selectivity. In contrast, the Fe@C-600 catalyst has higher CO conversion and a lower olefin/paraffin selectivity. For example, Fe@C-600 has an ethene-to-ethane ratio of 0.16, whereas for Fe@C-500, the ratio increases to 0.6. Similarly, higher ratios were obtained with higher carbon numbers; the O/P C_3_ ratio increased from 1.42 to 3.62, and the O/P C_4_ ratio from 2.12 to 3.76, respectively.

Unexpectedly, despite the lower X_CO_ of Fe@C-500, it shows comparable FTY and olefin yield with the more catalytically active Fe@C-600, as displayed in Fig. 6 and Table 3. The considerable FTY value of Fe@C-500 (660.8 mmol_CO_g^−1^_Fe_ h^−1^) could be attributed to several factors: the relatively lower Fe loading as depicted from AAS, the higher surface concentration of Fe as observed from XPS, and the better dispersion of Fe phase as seen from elemental mapping images. The second aspect is that olefin production potential (Selectivity and O/P) increases with a decrease in the pyrolysis temperature. This trend is reported by Luo et al. and Valero-Romero et al.^8,30^. This could be accounted for the higher surface oxygen content, as shown from XPS, that might mitigate the secondary hydrogenation of hydrocarbon products. This is verified by hydrogen chemisorption experiments showing the lower capability of Fe@C-500 for hydrogen adsorption.

Particle and pore size are other factors to consider. The impact of surface area and pore structure on product dispersion in FTS is well recognized^62–64^. The increased pore capacity of the catalysts allows for greater exposure of active metals during the FTS reaction and facilitates gas diffusion. The presence of bigger pores increases the likelihood of chain elongation and allows for better control of hydrogenation, resulting in higher levels of ${\text{S}}_{{\text{C}}_{5+}}$ and greater selectivity towards olefins^62,65^. For example, Fe@C-600 has a lower pore size (2.5 nm) than Fe@C-500 (6.3 nm), which may result in lower olefin selectivity and ${\text{S}}_{{\text{C}}_{5+}}$. Moreover, the Fe@C-500 catalyst combines the unique porous properties of MOF with the mesoporous structure of the pyrolyzed part, which may provide easier transport and lower secondary olefin hydrogenation^8^.

#### Evaluation of the FTS catalytic stability of the prepared Fe-BTC catalyst

**Fig. 8:**
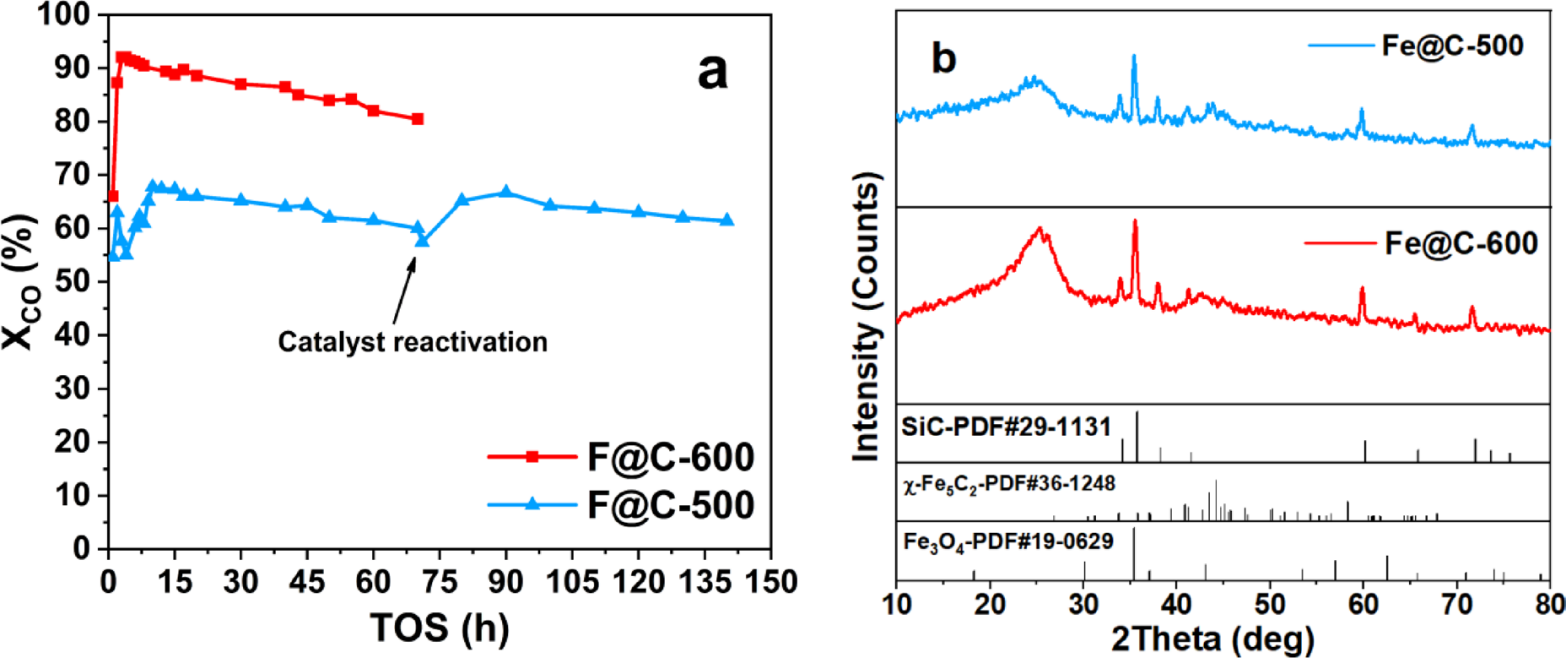
(**a**) Catalytic performance stability of catalysts at T = 340 °C, P = 20 bar, GHSV = 20,000 mL g^−1^_cat_ h^−1^, and H_2_/CO ratio of 1. (**b**) XRD of spent catalysts.

To check the stability of the catalyst, longer catalytic tests were performed for 70 h at T = 340 °C, P = 20 bar, GHSV = 20,000 mL g^−1^_cat_ h^−1^, and H_2_/CO ratio of 1, Fig. 8a. The catalysts show satisfactory stability for continuous TOS up to 70 h at a constant temperature of 340 °C and high GHSV of 20,000 mL g^−1^_cat_ h^−1^, Fig. 8a. Fe@C-500 exhibits a smaller decline in activity after 70 h (about 6%) in comparison to Fe@C-600 (approximately 10%). However, the activity could be restored to its starting value by catalyst reactivation under H_2_ flow at 400 °C for 2 h ^7^. The Fe@C-500 catalyst was reactivated after 70 h on stream, where the activity was back to its original value. The catalyst shows satisfactory stability for continuous TOS up to 140 h at a constant temperature of 340 °C and GHSV of 20,000 mL g^−1^_cat_ h^−1^. The noticed reduced stability, particularly common in systems with Fe particles exceeding 6 nm, is typically linked to ongoing chemical and structural alteration in the iron phases, plus the deposition of carbon on the catalyst surface^36,38^.

Figure 8b displays the XRD patterns for the spent catalysts. The primary phases present are Fe_3_O_4_ and χ-Fe_5_C_2_ (JCPDS file No. 19-0629 and No. 36-1248, respectively), along with SiC, which is used to dilute the catalyst. Weak peaks of iron carbide phase χ-Fe_5_C_2_ are shown with the presence of a substantial graphite peak at 25° in the XRD pattern of the spent Fe@C-600. Figure 8b provides evidence of observed less stability and deterioration of catalytic activity resulting from the deposition of carbon when compared with the more stable Fe@C-500. This is confirmed by the stronger typical peaks of the active iron carbide phase χ-Fe_5_C_2_ 43.4° and 44.1° of Fe@C-500.

#### Effect of operating conditions

**Fig. 9:**
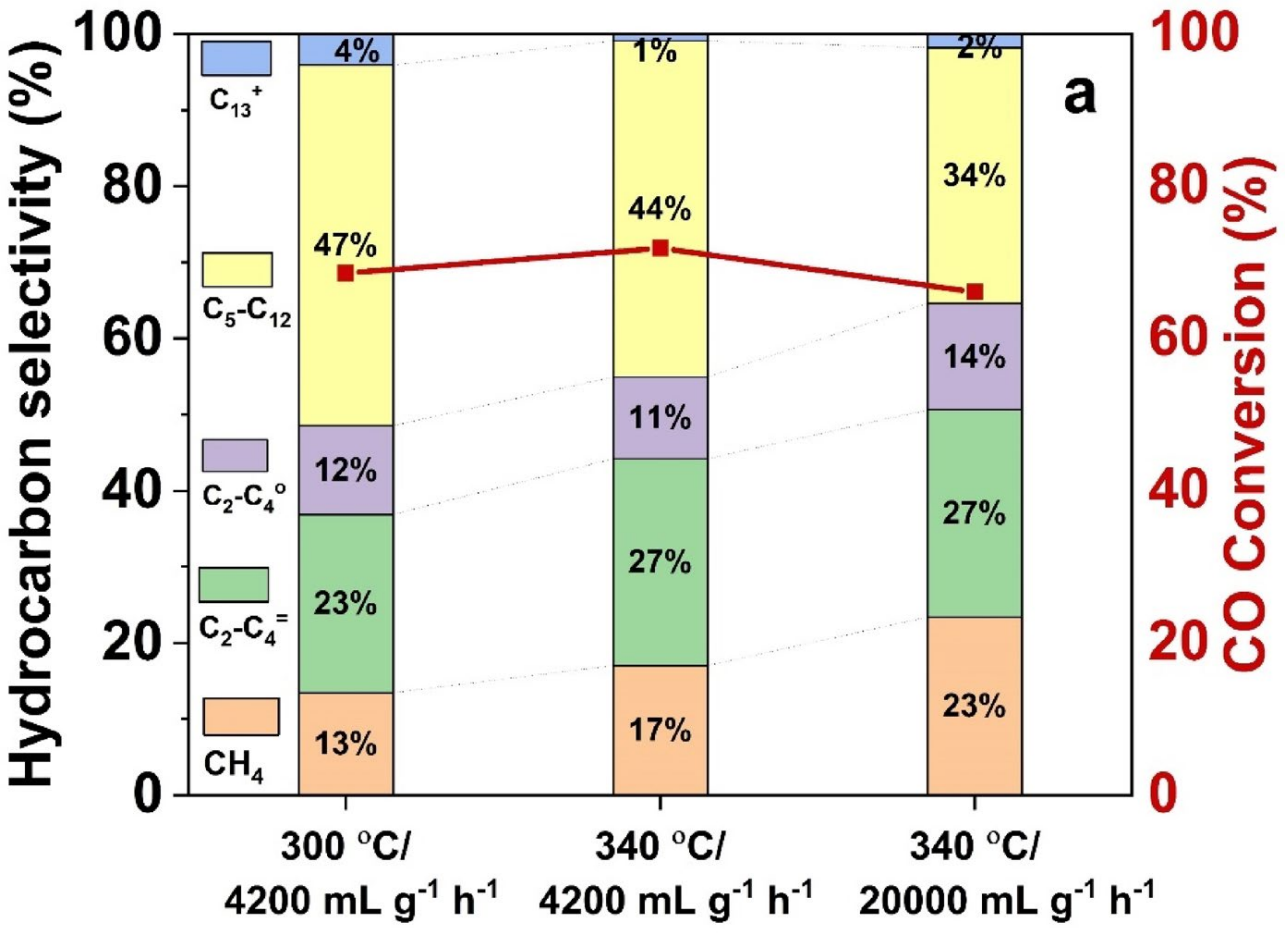
Product distribution comparison of Fe@C-500 at different temperatures and GHSV at P = 20 bar and H_2_/CO = 1.

Table 3 and Fig. 9 show how temperature and gas hourly space velocity (GHSV) affect olefin quality, product selectivity, and FTS activity. By raising the temperature from 300 to 340 °C, the CO conversion increased slightly (68.56–71.86%) at a constant GHSV of 4200 mL g^−1^_cat_ h^−1^. However, the methane (CH₄) selectivity increased (13.45–17.02%) and the C_5_ + selectivity decreased (51.45–45.05%), suggesting that cracking reactions are enhanced at higher temperatures. The findings show that heavier hydrocarbons are suppressed, whereas light olefin generation is favoured by increased temperature and gas hourly space velocity (GHSV). At a constant GHSV (4200 mL g^−1^_cat_ h^−1^), raising the temperature from 300 to 340 °C improved the selectivity of C_2_–C_4_ olefins (O/P ratio strengthened from 2.00 to 2.53) while decreasing C₅ + olefins (O/P dropped from 2.16 to 1.84). This implies that light olefin production is stimulated by higher temperatures because of improved cracking and dehydrogenation processes.

CO conversion reduced significantly (66.08%) when GHSV was boosted to 20,000 mL g^−1^_cat_ h^−1^ at 340 °C. At the same time, CH₄ and C₂–C₄ selectivity increased further (23.32% and 41.37%, respectively), while C₅ + selectivity declined substantially (35.30%). Shorter residence duration at greater GHSV promoted shorter hydrocarbon chains. The Fe-time yield (FTY) increased dramatically (660.8 mmol_CO_ g^−1^_Fe_ h^−1^) despite greater GHSV, at the expense of heavier hydrocarbon (C_5_^+^) and CO conversion, potentially because of improved mass transfer. C₂–C₄ olefin selectivity maintained its value (O/P = 1.95) when GHSV was raised to 20,000 mL g^−1^cat h⁻^1^ at 340 °C, while C₅ + olefins further decreased (O/P = 1.44). Interestingly, the FTY for light hydrocarbons increased (from 48.27–179.5 mmol_CO_ g^−1^_Fe_ h^−1^) while the yield of C₂–C₄ olefins stayed constant (9.25–9.90 mmol_CO_ g^−1^_Fe_ h^−1^). This suggests that higher GHSV increases light hydrocarbon productivity without compromising light olefin selectivity. These trends imply that higher temperature and GHSV cause the product distribution to shift towards lighter olefins, most likely as a result of shorter residence times and enhanced cracking.

### Comparison with prepared reference catalyst

**Fig. 10:**
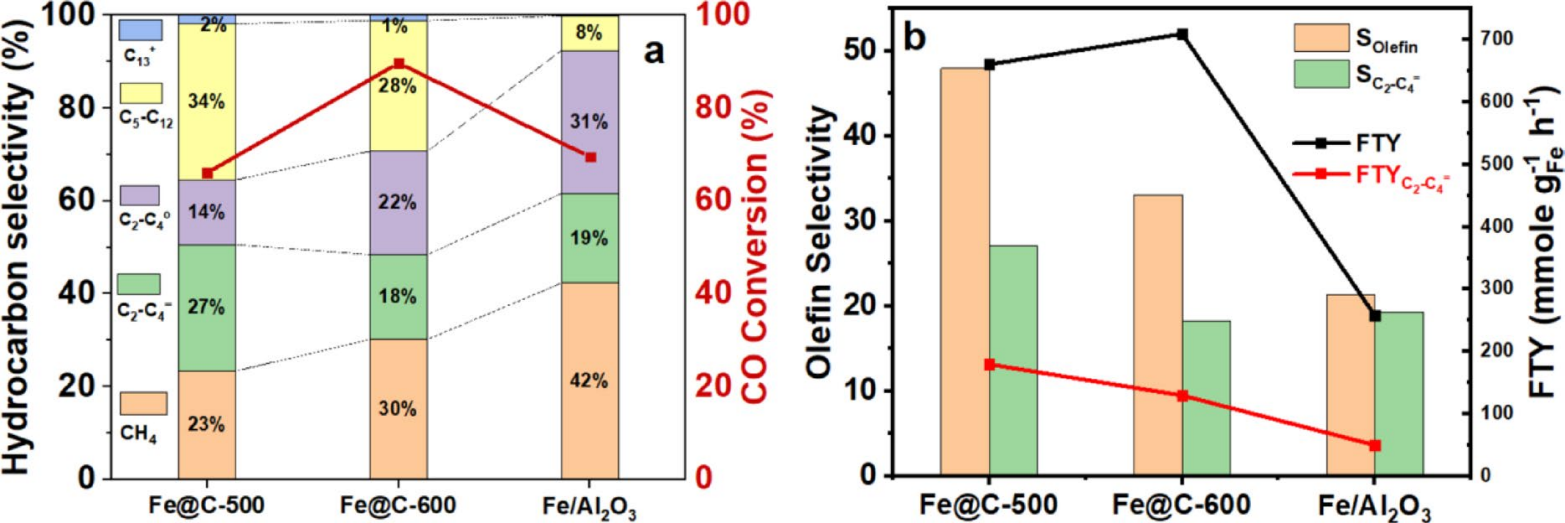
(**a**) Product distribution comparison and (**b**) olefin selectivity and FTY of Fe@C-500, Fe@C-600, and Fe/Al_2_O_3_, at T = 340 °C, P = 20 bar, GHSV = 20,000 mL g^−1^_cat_ h^−1^, and H_2_/CO = 1.

The IWI-synthesized Alumina supported catalyst (Fe/Al_2_O_3_) prepared in our previous study^7^ was tested as a reference catalyst at the same conditions, T = 340 °C, P = 20 bar, GHSV = 20,000 mL g^−1^_cat_ h^−1^, and H_2_/CO ratio of 1. Figure 10 highlights the diverse FTS behavior between the reference and prepared Fe-NDC-derived catalysts. The promoter-free Fe/Al_2_O_3_ catalyst has equivalent carbon monoxide conversion to that of Fe@C-500. However, Fe/Al_2_O_3_ has the highest methane (42.3%), carbon dioxide selectivity (50.5%), and light hydrocarbons (C_1_–C_4_ = 92.4%), Fig. 10(a and b) and Table 3. Furthermore, it has a smaller C_5+_ of 7.6% with α = 0.39. Besides, Fe/Al_2_O_3_ has the lowest total olefin selectivity/yield, O/P, FTY, and ${\text{FTY}}_{{\text{C}}_{2}-{{\text{C}}_{4}}^{=}}$, supplementary Fig. S4. However, it has a comparable light olefin selectivity (19.3%) and total paraffin selectivity (75.5%) to that of Fe@C-600. In contrast to Fe/Al_2_O_3_, promoter-free Fe-NDC catalysts exhibit enhanced results. The mismatch proves the beneficial impact of the carbon support of the Fe-NDC catalyst. The higher C_5+_ (29.3–35.3%) and gasoline-range hydrocarbons (C_5_–C_12_), ~ 30% for both catalysts, could be attributed to the porous carbon support.

### Comparison with previous work

Supporting Table S1 summarizes comparisons with other Fe-MOF-generated catalysts, references, and commercial catalysts published in the literature. Using various promoters and operating scenarios, such as gas GHSV and pressure, complicates the evaluation task. However, according to the table, the X_CO_ value for the prepared catalysts (66.1–89.7%) is similar to or greater than the reported Fe-MOF-derived catalysts. The overall Fe-time yield (660.8–709.7 mmol_CO_g^−1^_Fe_ h^−1^) and ${\text{FTY}}_{{\text{C}}_{2}-{{\text{C}}_{4}}^{=}}$ (129.6–179.5 mmol_CO_g^-1^_Fe_ h^-1^) are higher than most past studies, except for those that used higher GHSV values than 20,000 mL g^−1^_cat_ h^−1^.

The catalyst’s exceptional performance is highlighted when compared to the recognized Ruhrchemie industrial catalyst (Fe-Cu-K-SiO_2_)^66,67^, Table S1. The comparison shows that the Fe@C-500 catalyst, which was not promoted, has a lower X_CO_ but still maintains the same level of olefin selectivity/yield as the promoted Ruhrchemie catalyst. Additionally, the Fe@C-500 catalyst has higher total and light olefin FTY, as well as higher ${\text{S}}_{{\text{C}}_{5+}}$ values.

**Fig. 11:**
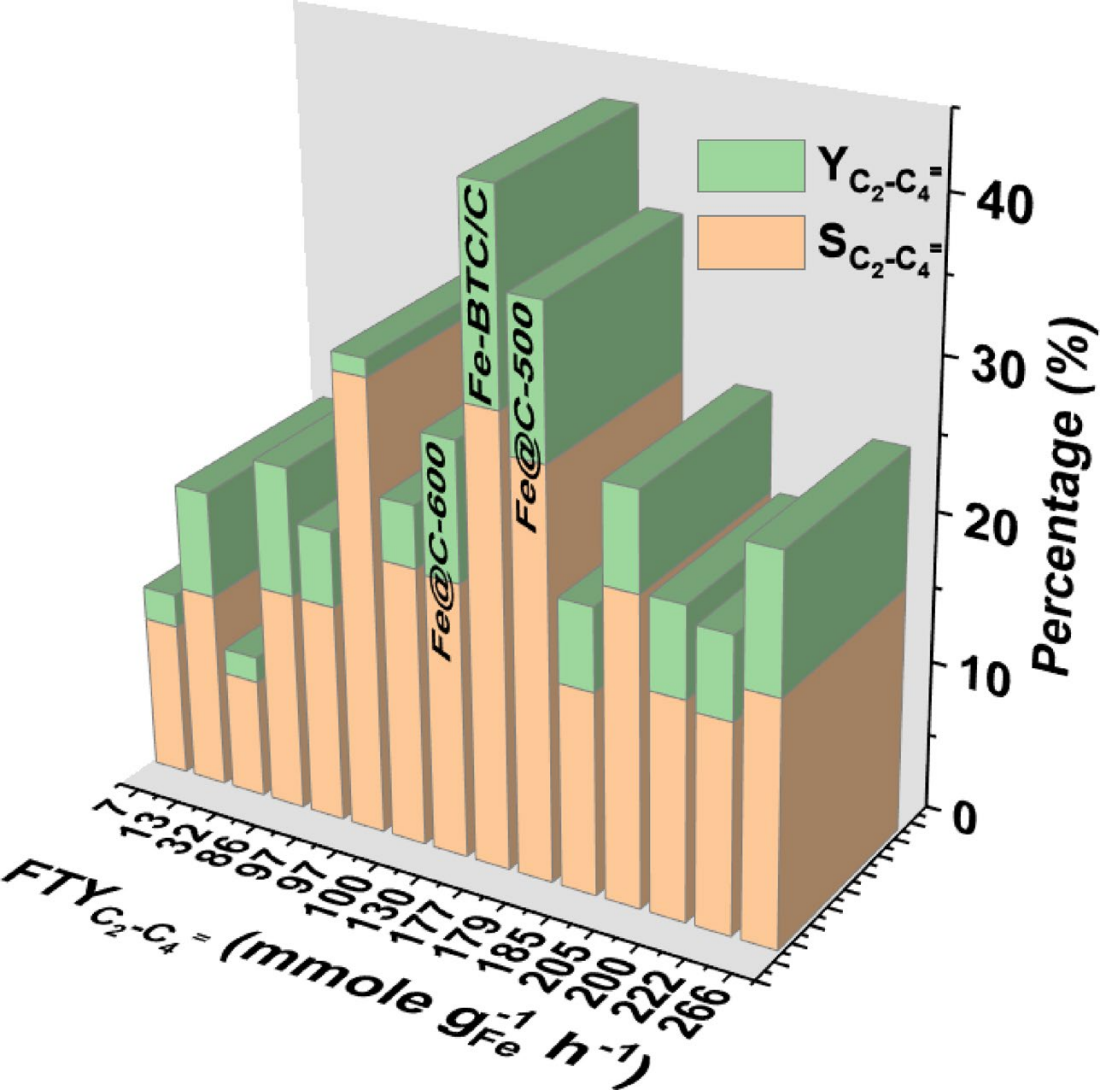
Olefin indicators produced in this work compared to state-of-the-art Fe-MOF-based catalysts.

Fe@C-500 exhibits notable performance in terms of total olefin selectivity (~ 50%) and light olefin yield and selectivity (17.5% and 27%, respectively). These values are either greater or equivalent to the majority of research, as shown in Table S1. Furthermore, it is equivalent to our previously documented Fe-BTC/C catalyst under the same conditions^7^, as shown in the Fig. 11. Furthermore, all experimental outcomes were achieved using catalysts without promoters, which boost the catalytic activity and olefin production.

Eventually, the Fe@C-500 catalyst described here demonstrates a superior combination of C_2_-C_4_ olefin selectivity, yield, and FTY, considering the current state of the art (see supplementary Table S1), exceeds a wide range of the existing literature^7,23,28,34,36–38,41,61,66–70^ (see Fig. 11). For example, the material significantly surpasses two of the recently published Fe-MOF catalysts (first and third row in Table S1), showing a C_2_–C_4_ olefins FTY ~ 27 and 6 times higher than the promoter-free Fe-MIL-88B and Fe-MIL-100-derived catalyst reported by Wang et al. and Qin et al., respectively^68,69^.

While this study reveals Fe@C-500’s better performance in selective olefin synthesis, various limitations should be noted. The investigation focused on only two pyrolysis temperatures (500 °C and 600 °C). The best temperature for balancing structural retention and activity was 500 °C. Lower temperatures (< 500 °C) were not examined due to potential negative effects such as excessive preservation of the MOF framework, which might mask active Fe sites and drastically reduce syngas conversion. Pure CO and green H_2_ were employed in experiments. However, real-world syngas from coal/biomass has variable H_2_/CO ratios while hosting other gases and impurities (e.g., H₂S) that might poison the catalyst, which was not explored in this work. Testing under authentic feedstock scenarios is required for evaluating catalytic performance. The catalyst’s performance was examined under controlled, short-term operating conditions (70 h). Continuous operation (> 100 h) can result in deactivation pathways (e.g., coking, sintering), as indicated by XRD of spent catalysts for Fe@C-600. The significant role of residual MOF porosity in suppressing olefin hydrogenation, although noticeable from selectivity trends, needs additional confirmation using in-situ spectroscopy or computational modelling.

## Conclusions

In summary, the Fe@C-500 catalyst produced from Fe-NDC doubles the olefin/paraffin ratio while retaining MOF-like porosity at 500 °C. The sustained structure of the catalyst allowed reactant movement and limited olefin hydrogenation, leading to an improved olefin/paraffin ratio. However, its untested stability for a prolonged time and limited for pure syngas warrant further optimization. Despite these limitations, the promoter-free Fe@C-500 catalyst’s light olefin selectivity and yield are more significant or equivalent to state-of-the-art Fe-MOF-derived catalysts. Additionally, it shows comparable olefin selectivity and yield, increased Fe-time yield for light olefins, and higher C_5+_ values compared to the promoted Ruhrchemie industrial catalyst. Going forward with practical applications, further research should examine long-term stability under a continuous flow of industrial syngas. These results establish Fe-NDC as a potential precursor for structure-controlled catalysts for converting syngas into valuable chemicals.

## Electronic supplementary material

Below is the link to the electronic supplementary material.

Supplementary Material 1

## Data Availability

All relevant data are available from the corresponding author at reasonable request.
