# Supplementary material for: Thermally stable metal–organic framework based iron 2,6-naphthalenedicarboxylic catalyst (Fe-NDC) for syngas conversion to olefin
Source: Sci Rep. 2025 Jul 22;15:26526. doi: 10.1038/s41598-025-09332-0 (PMC12280178; doi:10.1038/s41598-025-09332-0)
Supplement: Supplementary file 1 — Supplementary Material 1 [file 41598_2025_9332_MOESM1_ESM.docx]

**Thermally Stable Metal-Organic Framework based Iron 2,6-Naphthalenedicarboxylic Catalyst (Fe-NDC) for Syngas Conversion to Olefin**

Ahmed E. Rashed^1*^, Mohamed Nofal^2^, Ahmed Abd El-Moneim^3,4,5^

^1^ Environmental Sciences Department, Faculty of Science, Alexandria University, Alexandria, 21511, Egypt.

^2^ Institute of Graduate Studies and Research, Alexandria University, Alexandria, Egypt.

^3^ Graphene Center of Excellence, Egypt-Japan University of Science and Technology, New Borg El-Arab, 21934, Egypt.

^4^ Basic and Applied Science Institute, Egypt-Japan University of Science and Technology, New Borg El-Arab, 21934, Egypt.

^5^ Physical Chemistry Department, National Research Centre, El-Dokki, Cairo, 12622, Egypt.

^*^Corresponding author Email: ^*^envirashed@alexu.edu.eg

**Table of Contents**

**S1. Additional TGA and XPS characterization.**

**S2. Additional FTS performance results.**

**S1. Additional TGA and XPS characterization.**


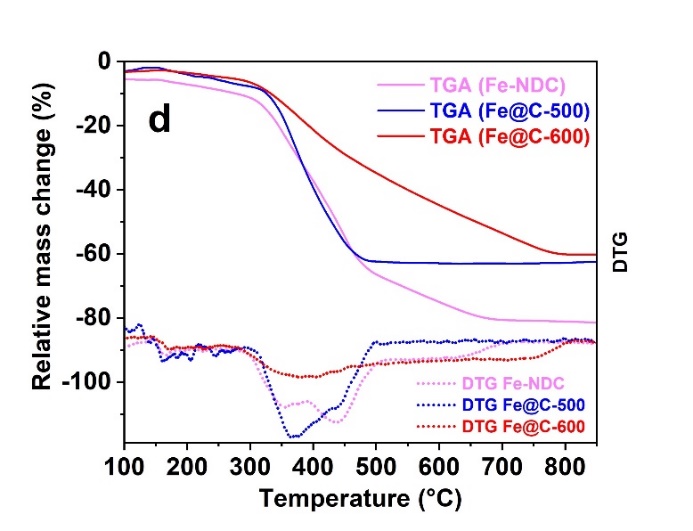


***Figure S1: TGA and DTG profiles of Fe-NDC and derived catalysts.***

| 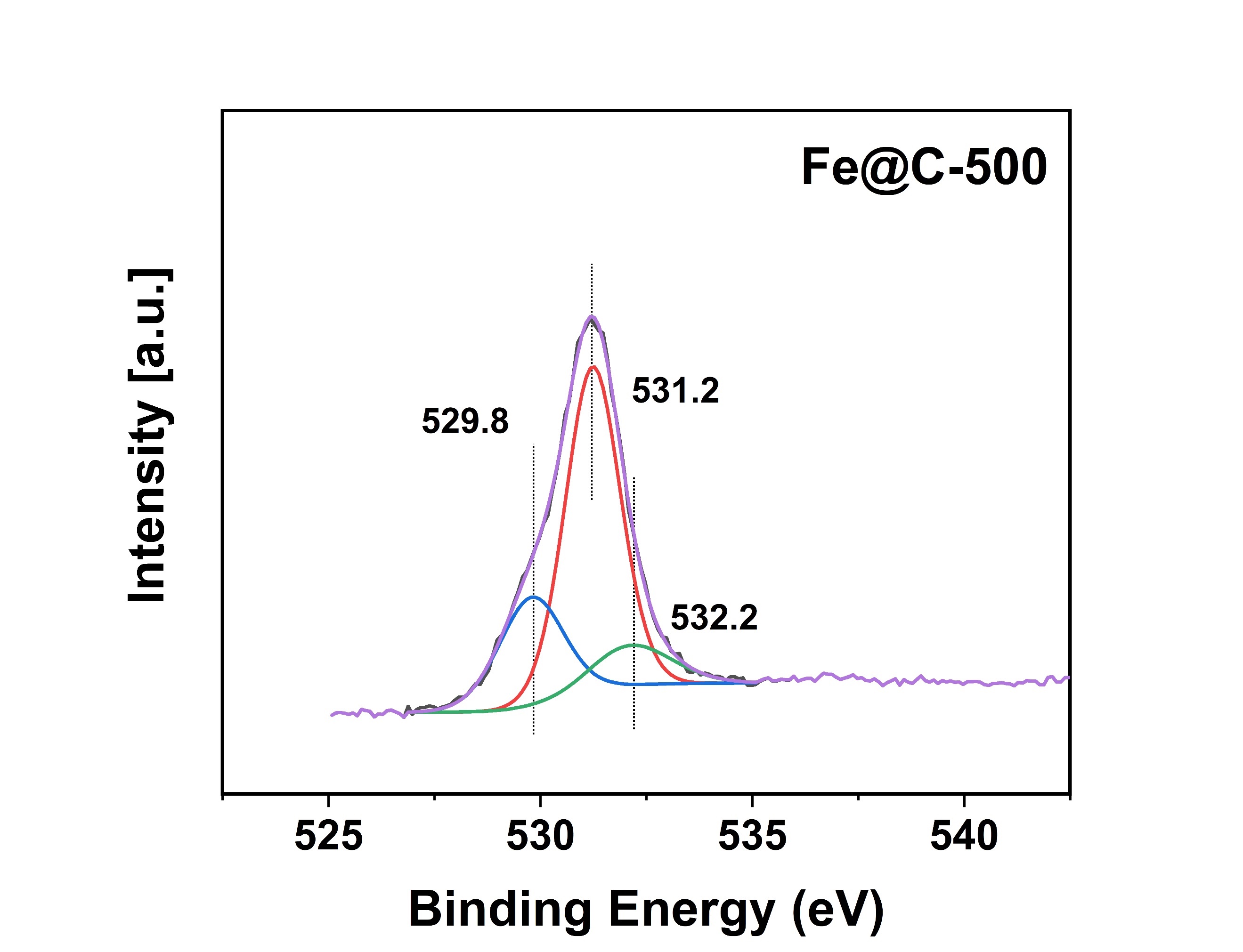 | 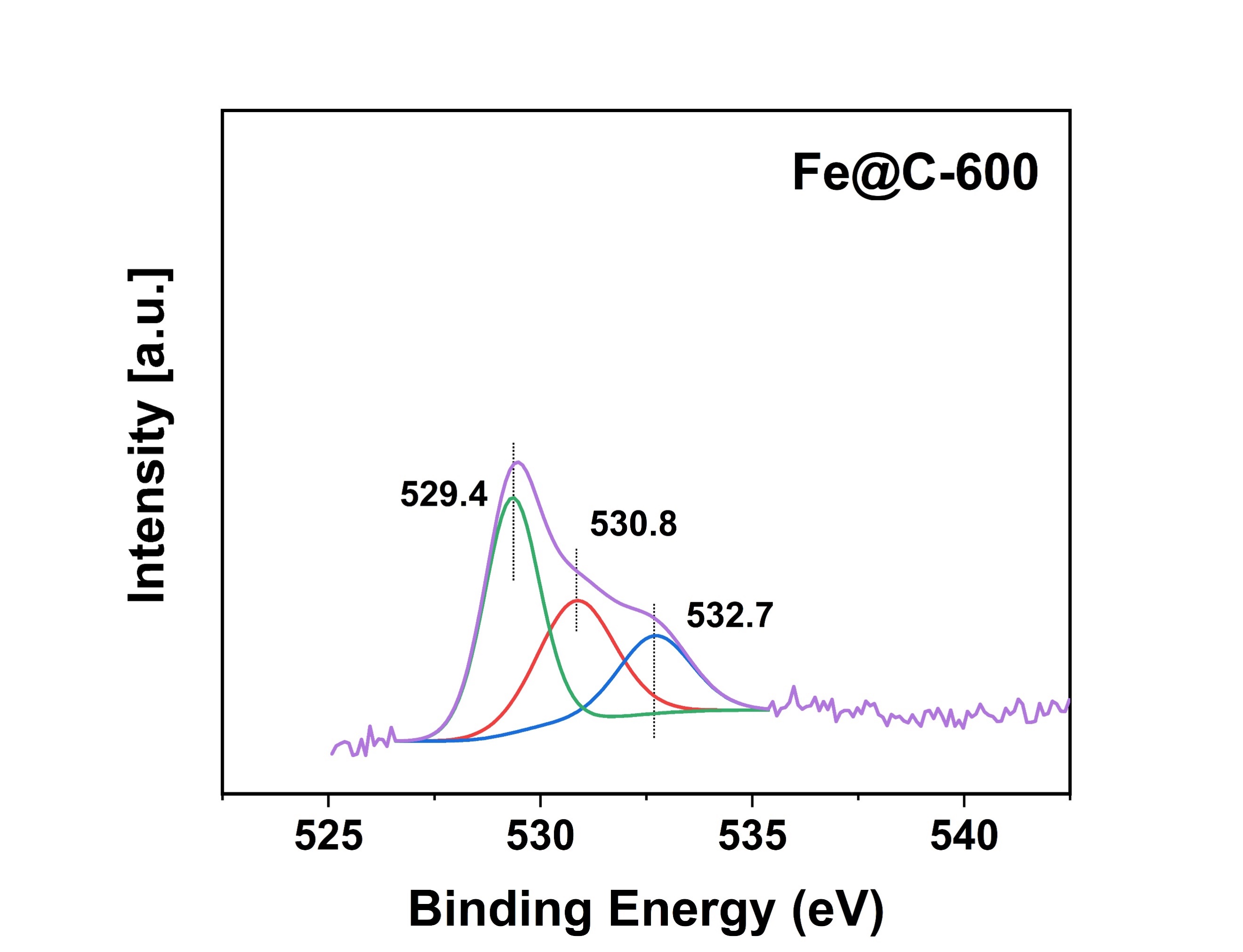 |
| --- | --- |
| **Figure S2: Os1 XPS spectra of (a) Fe@C-500 and Fe@C-600.** | |

**S2. Additional FTS performance results.**

| **a** | **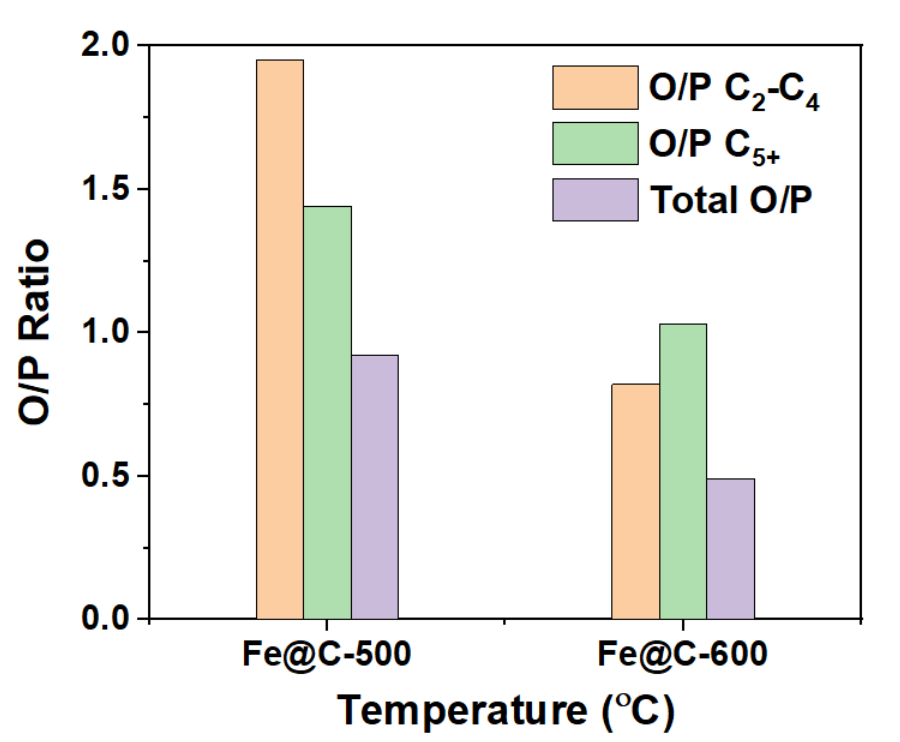** | **b** | **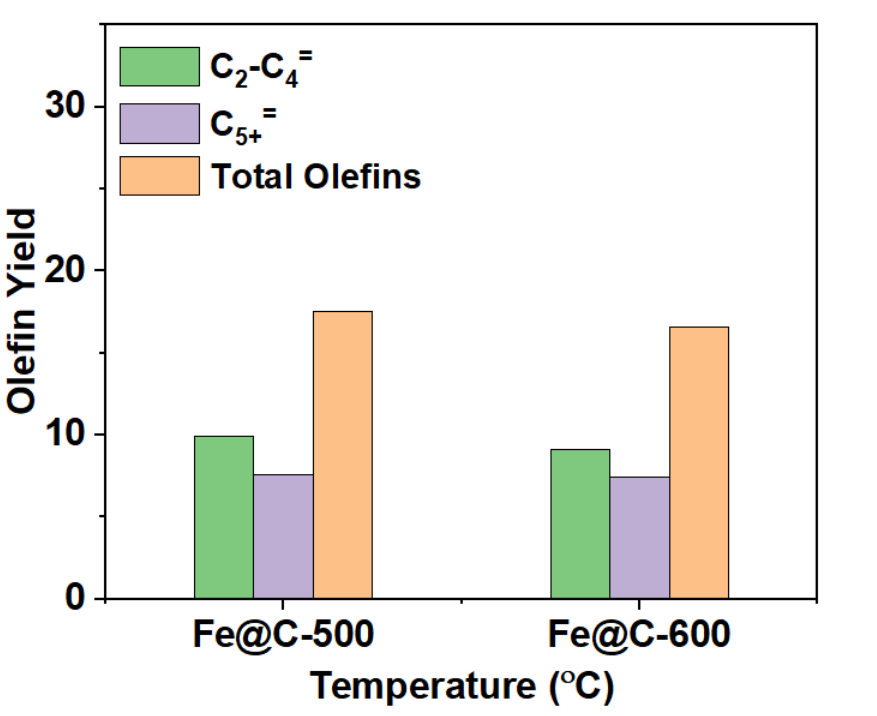** |
| --- | --- | --- | --- |
|  |  |  |  |
| **c** | 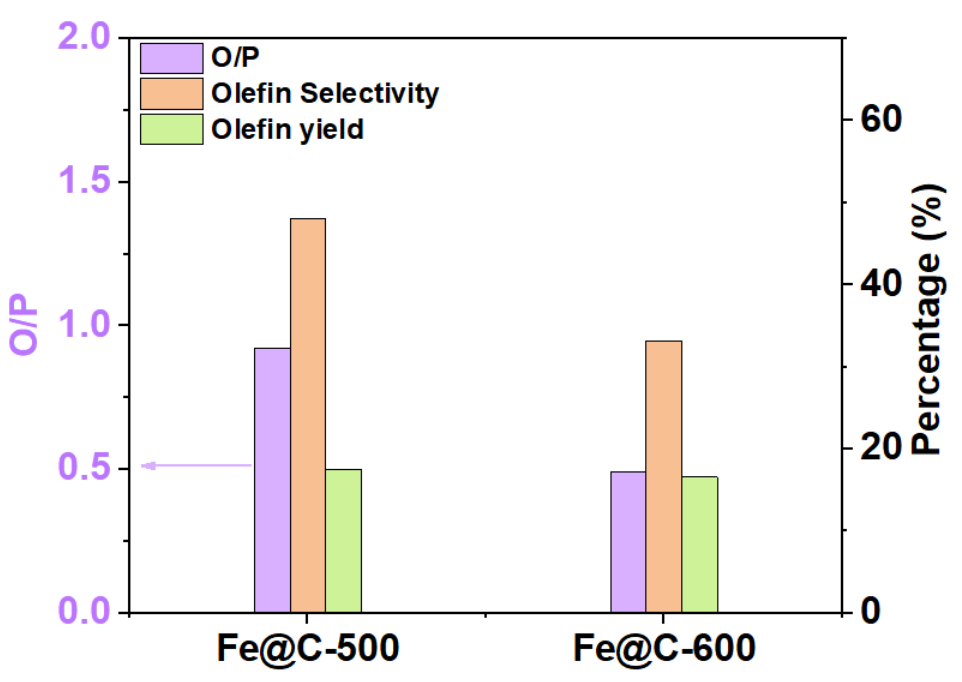 | **d** | 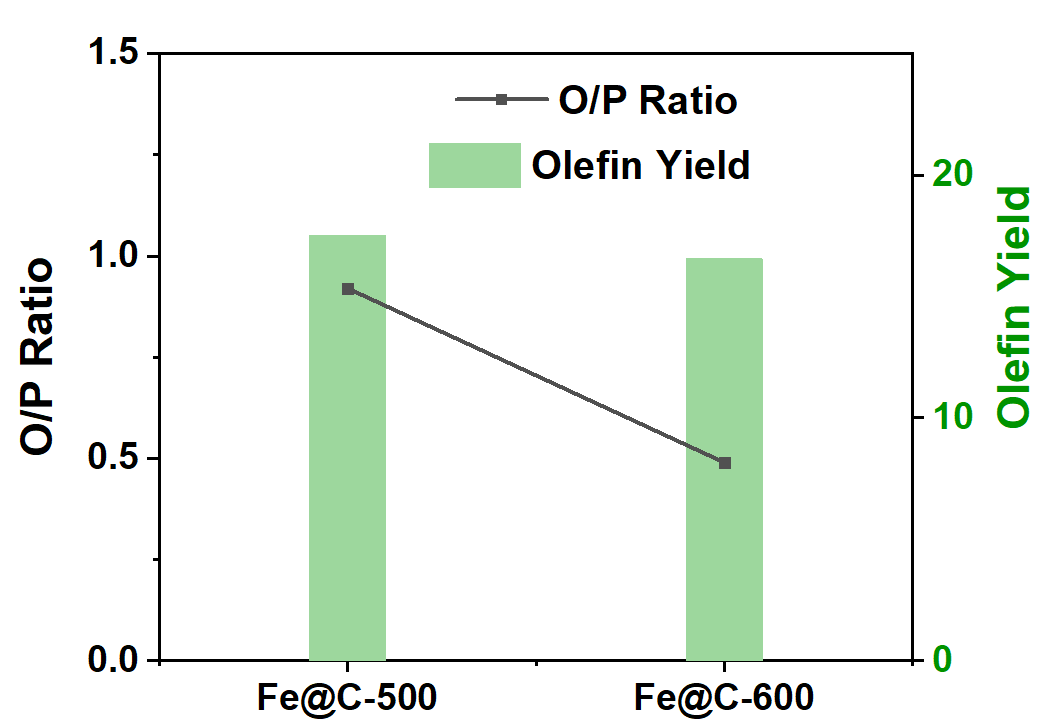 |

**Figure** **S3 Olefin yield, selectivety, and O/P ratio of derived catalysts.**


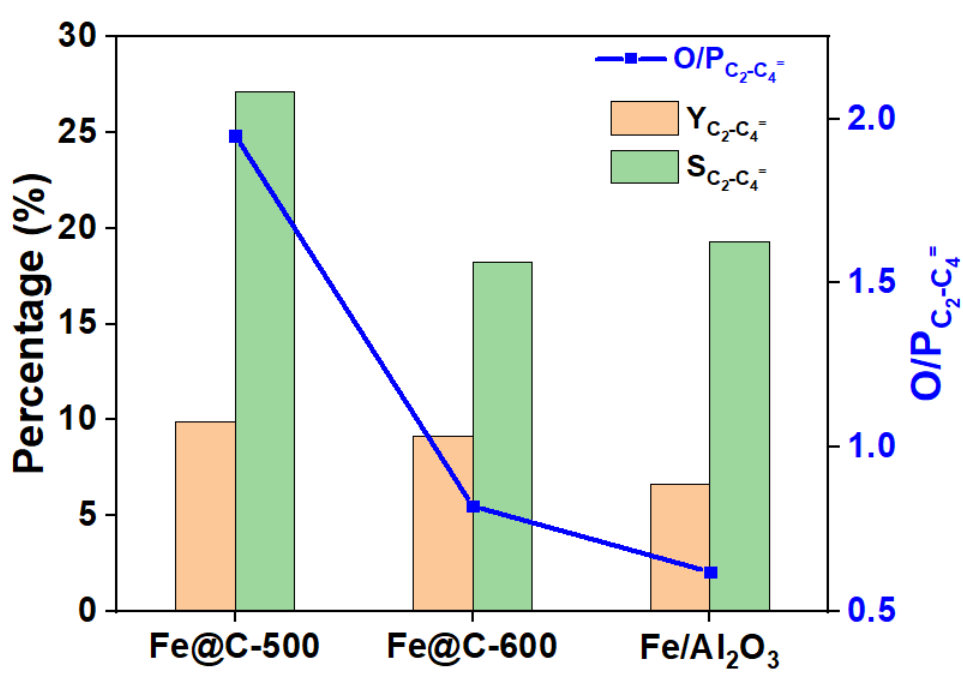


***Figure S3: comparison of olefin indicators for Fe@C-500, Fe@C-600, and reference catalyst (Fe/Al_2_O_3_), at T= 340 °C, P = 20 bar, GHSV = 20000 mL g^-1^_cat_ h^-1^, and H_2_/CO = 1.***

| Table S1: FTS performance of the state-of-the-art promoter-free Fe-MOF-derived and industrial catalysts. | | | | | | | | |
| --- | --- | --- | --- | --- | --- | --- | --- | --- |
| Ref. | **Catalyst (MOF precursor)** | **Reaction conditions:**  **P(bar), T(°C), H_2_/CO, GHSV (mL g^-1^_cat_ h^-1^)** | $\mathbf{X}_{\mathbf{CO}}$ **(%)** | **S_C5+_** | $\mathbf{S}_{\mathbf{C}_{\mathbf{2}}\mathbf{-}\boldsymbol{C}_{\boldsymbol{4}}^{\boldsymbol{=}}}$ **(%)** | $\mathbf{Y}_{\mathbf{C}_{\mathbf{2}}\mathbf{-}\boldsymbol{C}_{\boldsymbol{4}}^{\boldsymbol{=}}}$ **(%) ^a^** | **FTY (mmol_CO_g^-1^_Fe_ h^-1^)** | $\mathbf{FTY}_{\mathbf{C}_{\mathbf{2}}\mathbf{-}\boldsymbol{C}_{\boldsymbol{4}}^{\boldsymbol{=}}}$ **(mmol g^-1^_Fe_ h^-1^)** |
| ^1^ | Fe@C  (MIL-100) | 25, 340, 1, 5000 | 27 | 63 | 10.2 | 2.2 | 65 | 6.6 |
| ^2^ | Fe-MIL-88B-T/C | 20, 340, 1, 4200 | 96 | 22 | 13 | 7 | 102.1 | 13.2 |
| ^3^ | Fe@C  (Fe-MIL-88B) | 20, 300, 1, 36000 | 23.5 | 85 | 7.9 | 1.7 | 40.3.2 | 31.8 |
| ^4^ | Fe@C-500 (Fe-MIL-100) | 30, 260, 2, 8000 ^b^ | 68 | 71.6 | 14.5 | 8.5 | 590.4 | 85.6 |
| ^5^ | MIL-101-7 W | 20, 340, 1, 13300 ^b^ | 49.8 | 35 | 14.5 | 5 | 666 | 96.5 |
| ^6^ | Fe/C-Aero | 10, 340, 2, 48000 | 7.5 | 5 | 30 | 1.2 | 324 | 97.2 |
| ^7^ | Fe-MIL-88B/C | 20, 300, 1, 36000 | 33.8 | 63.4 | 18.5 | 4.1 | 540 | 99.9 |
| This work | **Fe@C-600** | **20, 340, 1, 20000** | **89.7** | **29.3** | **18.3** | **9.1** | **709.7** | **129.6** |
| ^8^ | Fe-BTC/C | 20, 340, 1, 20000 | 86 | 39 | 29.8 | 13.5 | 523.5 | 176.8 |
| This work | **Fe@C-500** | **20, 340, 1, 20000** | **66.1** | **35.3** | **27.2** | **9.9** | **660.8** | **179.5** |
| ^9^ | 38Fe@C (Basolite F300) | 15, 340, 1, 55 ^c^ | 70 | 14 | 13.5 | 5.5 | 1368 | 184.6 |
| ^10^ | Fe/CNS(1000) (ZIF-8) | 10, 340, 1, 9000 | 45.9 | 39.2 | 20.5 | 6.5 | 1000.8 | 205.1 |
| ^11^ | 38-Fe@C (Basolite F300) | 20, 340, 1, 60000 | 77 | 14 | 14.6 ^d^ | 6.0 ^e^ | 1368 | 199.7 |
| ^12^ | Fe@C-500 (Basolite F300) | 20, 340, 1, 60000 | 76 | ---- | 14 ^d^ | 5.5 ^e^ | 1584 | 221.7 |
| ^3^ | Fe@C@SiO_2_-2 (Fe-MIL-88B) | 20, 300, 1, 36000 | 88.7 | 70.6 | 16.3 | 9.1 | 1634.4 | 266.4 |
| Reference and commercial catalysts | | | | | | | | |
| This work | Fe/Al_2_O_3_ | 20, 340, 1, 20000 | 69.5 | 7.2 | 19.3 | 6.6 | 257.7 | 49.7 |
| ^10^ | Fe/AC | 10, 340, 1, 9000 | 7.7 | 62.3 | 14.3 | 1 | 205.2 | 29.3 |
| ^10^ | Fe/CP (ZIF-8)^f^ | 10, 340, 1, 18000 | 10.1 | 28.5 | 31.2 | 2.5 | 471.6 | 147.1 |
| ^13^ | Ruhrchemie (Fe-Cu-K-SiO_2_) | 20, 340, 1, 1500 ^b^ | 79 | 18 | 36 | 17.9 | 40.3 | 14.5 |
| ^14^ | Ruhrchemie (Fe-Cu-K-SiO_2_) | 15, 250, 0.67, 2000 | 64.4 | 65.4 | 15.66 | -- | 209.5^g^ | 32.8 |

^a^ The olefin yield corrected for CO_2_ and unreacted CO. $Y_{C_{2}-C_{4}^{=}} (\%)=\frac{S_{C_{2}-C_{4}^{=}}*X_{\mathrm{CO}}*(100-S_{\mathrm{CO}_{2}})}{100*100}$ , ^b^ Unit: h^-1^, ^c^ Unit: mmol_CO, STP_ g^-1^_Fe_ min^-1^, ^d^ $S_{C_{2}-C_{5}^{=}}$ ^e^ $Y_{C_{2}-C_{5}^{=}}$, ^f^ CP carbon particles obtained by calcining the commercial ZIF-8, ^g^ Unit: mmol_CO+H2,_ g^-1^_Fe_ h^-1^_._

**References**

1 A. Wang, M. Luo, B. Lü, Y. Song, M. Li and Z. Yang, *Molecular Catalysis*, 2021, **509**, 111601.

2 A. E. Rashed, A. Nasser, M. F. Elkady, Y. Matsushita and A. A. El-Moneim, *ACS Omega*, 2022, **7**, 8403–8419.

3 H. Qin, Y. Zhou, Q. Huang, Z. Yang, R. Dong, L. Li, J. Tang, C. Zhang and F. Jiang, *ACS Appl Mater Interfaces*, 2021, **13**, 5460–5468.

4 X. Yang, X. Guo, C. Zhang, X. Wang, Y. Yang and Y. Li, *Acta Chimi Sin*, 2017, **75**, 360–366.

5 Y. Wu, Z. Huang, H. Jiang, C. Wang, Y. Zhou, W. Shen, H. Xu and H. Deng, *ACS Appl Mater Interfaces*, 2019, **11**, 44573–44581.

6 M. Oschatz, S. Krause, N. A. Krans, C. Hernández Mejía, S. Kaskel and K. P. De Jong, *Chemical Communications*, 2017, **53**, 10204–10207.

7 B. An, K. Cheng, C. Wang, Y. Wang and W. Lin, *ACS Catal*, 2016, **6**, 3610–3618.

8 A. E. Rashed, M. F. Elkady, Y. Matsushita, A. Nasser and A. Abd El-Moneim, *Chemical Engineering Journal*, 2023, **473**, 145125.

9 L. Oar-Arteta, M. J. Valero-Romero, T. Wezendonk, F. Kapteijn and J. Gascon, *Catal Sci Technol*, 2018, **8**, 210–220.

10 Q. Zhao, S. Huang, X. Han, J. Chen, J. Wang, A. Rykov, Y. Wang, M. Wang, J. Lv and X. Ma, *Carbon N Y*, 2021, **173**, 364–375.

11 V. P. Santos, T. A. Wezendonk, J. J. D. Jaén, A. I. Dugulan, M. A. Nasalevich, H. U. Islam, A. Chojecki, S. Sartipi, X. Sun, A. A. Hakeem, A. C. J. Koeken, M. Ruitenbeek, T. Davidian, G. R. Meima, G. Sankar, F. Kapteijn, M. Makkee and J. Gascon, *Nat Commun*, 2015, **6**, 6451.

12 T. A. Wezendonk, V. P. Santos, M. A. Nasalevich, Q. S. E. Warringa, A. I. Dugulan, A. Chojecki, A. C. J. Koeken, M. Ruitenbeek, G. Meima, H. U. Islam, G. Sankar, M. Makkee, F. Kapteijn and J. Gascon, *ACS Catal*, 2016, **6**, 3236–3247.

13 H. M. Torres Galvis, J. H. Bitter, C. B. Khare, M. Ruitenbeek, A. I. Dugulan and K. P. De Jong, *Science (1979)*, 2012, **335**, 835–838.

14 W. Ma, Y. Ding, V. H. C. Vázquez and D. B. Bukur, 2004, **268**, 99–106.
